# Design, Synthesis, and Anticancer Evaluation of Novel Indole Derivatives of Ursolic Acid as Potential Topoisomerase II Inhibitors

**DOI:** 10.3390/ijms21082876

**Published:** 2020-04-20

**Authors:** A-Liang Li, Yun Hao, Wen-Yan Wang, Qing-Song Liu, Yue Sun, Wen Gu

**Affiliations:** Jiangsu Provincial Key Lab for the Chemistry and Utilization of Agro-Forest Biomass, Jiangsu Key Lab of Biomass-Based Green Fuels and Chemicals, Co-Innovation Center for Efficient Processing and Utilization of Forest Products, College of Chemical Engineering, Nanjing Forestry University, Nanjing 210037, China; lialiang673@gmail.com (A-L.L.); nfuhg2209@gmail.com (Y.H.); wwy1914898594@gmail.com (W.-Y.W.); liuqings673@gmail.com (Q.-S.L.); sunyue0620@gmail.com (Y.S.)

**Keywords:** ursolic acid, indole derivatives, anticancer activity, topoisomerase II, apoptosis

## Abstract

In this study, a series of new indole derivatives of ursolic acid bearing different *N*-(aminoalkyl)carboxamide side chains were designed, synthesized, and evaluated for their in vitro cytotoxic activities against two human hepatocarcinoma cell lines (SMMC-7721 and HepG2) and normal hepatocyte cell line (LO2) via MTT assay. Among them, compound **5f** exhibited the most potent activity against SMMC-7721 and HepG2 cells with IC_50_ values of 0.56 ± 0.08 μM and 0.91 ± 0.13 μM, respectively, and substantially lower cytotoxicity to LO2 cells. A follow-up enzyme inhibition assay and molecular docking study indicated that compound **5f** can significantly inhibit the activity of Topoisomerase IIα. Further mechanistic studies performed in SMMC-7721 cells revealed that compound **5f** can elevate the intracellular ROS levels, decrease mitochondrial membrane potential, and finally lead to the apoptosis of SMMC-7721 cells. Collectively, compound **5f** is a promising Topoisomerase II (Topo II) inhibitor, which exhibited the potential as a lead compound for the discovery of novel anticancer agents.

## 1. Introduction

Cancer has become a major cause of death in human beings. In 2018, there were estimated 18.1 million new cancer cases and 9.6 million cancer deaths worldwide [1]. In China, about 3.8 million new cancer cases and 2.29 million cancer deaths occurred in 2018, both ranked first among countries worldwide [1]. Despite their indispensable role in the treatment of cancer, chemotherapies often cause less satisfactory results due to severe side effects and rapidly occurring drug resistance [2]. Therefore, efforts to develop low-toxic and high-efficacy anticancer agents have still been the primary task of medicinal chemists.

Topoisomerase, which are generally classified as types I and II, are critical nuclear enzymes essential for cell proliferation by solving the topological hurdles in the process of DNA replication. Topoisomerase I (Topo I) handles the breaking and rejoining of only a single strand of a DNA duplex to relieve the topological tension. In the case of topoisomerase II (Topo II), it relaxes the DNA double helices by scissoring and religating both strands [3,4,5]. The monomeric enzyme Topo I does not require Mg(II) for DNA relaxation, while homodimeric enzyme Topo II requires Mg(II) and ATP hydrolysis for the enzyme’s function and rapid kinetics [3]. The Topo II family has two isozymes—Topo IIα and IIβ. Only the level of Topo IIα protein among human topo isozymes (Topo I, Iiα, and IIβ) increases in proliferative stage dependent manner. In addition, Topo II is frequently overexpressed in different types of tumors, so the expression level of Topo IIα has been used as a useful diagnostic marker for chemotherapy in diverse cancers [6,7]. These findings provide insight that targeting Topo II, especially Topo IIα, can be a good strategy for aiming at actively proliferating cancer cells rather than normal cells. A number of Topo II-targeted chemotherapy drugs, such as etoposide (VP-16), doxorubicin (DOX), and mitoxantrone have been approved by the FDA for clinical use [8]. However, most Topo II inhibitors exhibited rebarbative side effects including cardiotoxicity, development of secondary malignancies and multidrug resistance [9]. Hence, it is still a necessity to develop novel Topo II inhibitors with high efficacy and selectivity.

Natural products have long been used to treat various diseases due to their wide range of pharmacological activities [10]. Since the structural modified derivatives of natural products with anticancer activities generally have fewer side effects, a simple and effective method to obtain anticancer agents is the structural modification of bioactive natural products [11]. Ursolic acid (UA, **1**) is a natural pentacyclic triterpenoid abundant in many vegetables, fruits, and herbs. The compound and its derivatives exhibit a wide variety of biological activities, such as antibacterial [12], anticancer [13], antiviral [14], anti-inflammatory [15], antidiabetic [16], antiulcer [17], antiarrhythmic [18], anti-osteoporosis [19], anti-atherosclerotic [20], and anti-neurodegenerative activities [21]. In addition, some pentacyclic triterpenoids including UA were reported to inhibit Topo I and IIα by contending with DNA for topoisomerase binding sites, thus preventing topoisomerase-DNA cleavable complex formation [22,23,24]. Therefore, UA could be a promising starting material for the discovery of new topoisomerase inhibitors as anticancer agents.

**Figure 1:**
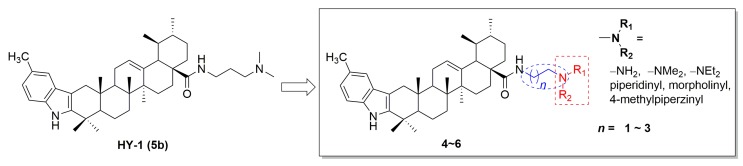
The structural modification of HY-1 (**5b**).

In an effort to discover novel anticancer agents, we have previously designed and synthesized a series of indole derivatives of UA bearing *N*-aminoalkyl side chains. In the in vitro cytotoxic assay, some derivatives showed significant cytotoxic activity against two human hepatocarcinoma cell lines (SMMC-7721 and HepG2). Among them, HY-1 (compound **5b**) exhibited the strongest activity with IC_50_ values of about 1 μM [25]. Moreover, in a preliminary screening, HY-1 showed considerable inhibitory activity to Topo I and IIα. Based on these findings, this compound was chosen as the starting material for further structure modifications. Since, in our preliminary study, the carbon chain and *N*-containing moiety of C-28 side chain played an important role in the cytotoxic activity of derivatives, we focused on the modification of the side chain in this work (Figure 1). As a result, a series of new UA derivatives were designed and synthesized in order to find derivatives with better anticancer activities. Furthermore, the anticancer mechanisms of the active compounds were also extensively explored.

## 2. Results

### 2.1. Chemistry

**Scheme 1:**
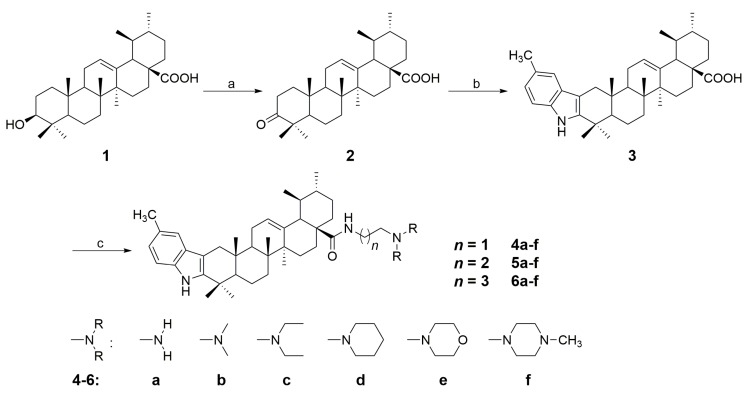
Synthetic route for the indole derivatives (**4a**–**f**, **5a**–**f**, **6a**–**f**) from ursolic acid. Reagents and conditions: (**a**) Jones reagent, acetone, 0 °C, 5 h; (**b**) *p*-methyl phenylhydrazine hydrochloride, EtOH, conc. HCl, reflux for 3 h; (**c**) *N*,*N*-dialkylethane-1,2-diamine, *N*,*N*-dialkylpropane-1,3-diamine, or *N*,*N*-dialkylbutane-1,4-diamine (1.2 eq), HOBt, DCC, CH_2_Cl_2_, rt, 12 h.

**Scheme 2:**
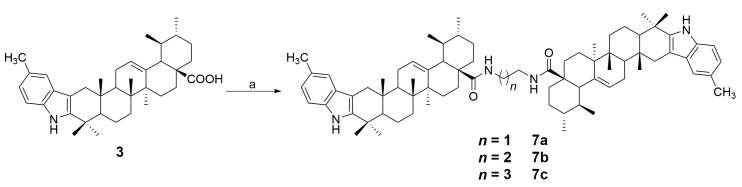
Synthesis of compounds **7a**–**c** from **3**. Reagents and conditions: (**a**) 1,2-ethylenediamine, 1,3-propanediamine and 1,4-butanediamine (0.5 eq), HOBt, DCC, CH_2_Cl_2_, rt, 12 h.

Based on the method reported earlier [25], a series of new indole derivatives of ursolic acid with different amine side chains were synthesized. Synthetic routes for the target compounds were summarized in Scheme 1 and Scheme 2. Briefly, the starting material UA (**1**) was oxidized by Jones reagent to afford 3-oxo-ursolic acid (**2**) in 76% yield. Subsequently, compound **2** was converted to the indole derivative (**3**) in 69% yield by reacting with *p*-tolylhydrazine hydrochloride through Fisher indole reaction. Further, compound **3** was reacted with different *N*^1^,*N*^1^-dialkyl-1,2-ethanediamine, *N*^1^,*N*^1^-dialkyl-1,3-propanediamine, or *N*^1^,*N*^1^-dialkyl-1,4-butanediamine to afford the corresponding amide derivatives **4a**–**f**, **5a**–**f**, and **6a**–**f** in 72–93% yields (Scheme 1). Interestingly, when compound **3** was reacted with 1,2-ethanediamine, 1,3-propanediamine, or 1,4-butanediamine in the presence of HOBt and DCC, the dimeric byproducts (**7a**–**c**) could always be detected. However, if we treat compound **3** with 0.5 eq of 1,2-ethanediamine, 1,3-propanediamine or 1,4-butanediamine, the dimeric derivatives **7a**–**c** became the main products with the yield of 61%, 65%, 63%, respectively (Scheme 2). All the synthesized compounds were purified by recrystallization or silica gel column chromatography, and the structures of these compounds were characterized through their HRMS, IR, ^1^H NMR and ^13^C NMR spectra (^1^H and ^13^C NMR spectra: Figure S1–S42 in the Supplementary Materials).

### 2.2. Cytotoxic Assay

The in vitro cytotoxic activities of synthesized compounds were evaluated against two human hepatocarcinoma cell lines (SMMC-7721 and HepG2) and the human normal hepatocyte cell line (LO2) via MTT assay. All tested compounds were dissolved in DMSO and the stock solutions were diluted by DMEM medium before treatment of the cultured cells. The IC_50_ values of the tested compounds against four cell lines were shown in Table 1.

As shown in Table 1, most synthesized compounds showed considerable cytotoxic activity against two cancer cell lines. Among them, compounds **4d**, **4f**, **5b**–**d**, **5f**, **6a** and **6f** displayed strong inhibition with IC_50_ values of around 1 μM. Especially, compound **5f** exhibited the most potent anticancer activity with IC_50_ values of 0.56 ± 0.08 and 0.91 ± 0.13 μM, respectively, which were comparable to those of reference drugs. Notably, compound **5f** showed significantly lower cytotoxicity to human normal hepatocyte cell line LO2 (IC_50_: 10.58 ± 0.52 μM), indicating that the compound had good selectivity between hepatocarcinoma and noncancerous hepatocyte cells. Compounds **5d** and **6f** also exhibited significant activities against SMMC-7721 cells with IC_50_ values of 0.89 ± 0.11 and 0.65 ± 0.07 μM, respectively. In addition, compounds **4a**–**c**, **4e**, **5a**, **5e**, and **6b**–**e** showed good inhibitory activity to the two cancer cell lines. On the contrary, compounds **7a**–**c** were inactive to all three cell lines (IC_50_ > 50 μM).

The results indicated that the anticancer activities of the synthesized compounds were affected by the *N*-containing moieties anchored on the side chain. For Series **4a**–**f**, **5a**–**f**, and **6a**–**f**, compounds **4f**, **5f**, and **6f** with *N*-methylpiperazine moieties all exhibited the strongest activities in their own series. Compounds **4a**–**d**, **5a**–**d**, and **6a**–**d** containing NH_2_, N(Me)_2_, N(Et)_2_, and piperidine moieties generally showed strong activities. However, compounds **4e**, **5e**, and **6e** with morpholine rings displayed relatively weaker inhibition to cancer cells compared with their analogs in the same series. In general, the order of effects to the cytotoxicity of these derivatives could be summarized as *N*-methylpiperazine > NH_2_, N(Me)_2_, N(Et)_2_ and piperidine > morpholine. On the other hand, the length of amide side chain also affected the cytotoxicity to some extent. Generally, compounds **5af** exhibited stronger cytotoxic activities than compounds **4a**–**f** and **6a**–**f**, which suggested that the 3C alkyl linker could be more beneficial to the cytotoxicity than 2C and 4C alkyl linkers. In addition, three dimeric derivatives **7a**–**c** were all inactive to tested cancer cells (IC_50_ > 50 μM), which indicated that such dimerization by alkyl linkers might hinder the interaction between these compounds and their potential targets, thus significantly decreasing their anticancer activities.

### 2.3. Topoisomerase Inhibition Assay

The conversion of supercoiled pBR322 plasmid DNA to relaxed DNA by Topo I and II was examined in the presence of the prepared compounds **4a**–**f**, **5a**–**f**, **6a**–**f**, and **7a**–**c**. Camptothecin and etoposide, well-known Topo I and II inhibitors, respectively, were used as positive controls. The effect of the tested compounds on Topo I and II activities were evaluated by Topo I/II relaxation assays. The reaction products, relaxed DNA could be distinguished from supercoiled DNA by gel electrophoresis and visualized using gel imaging system.

**Figure 2:**
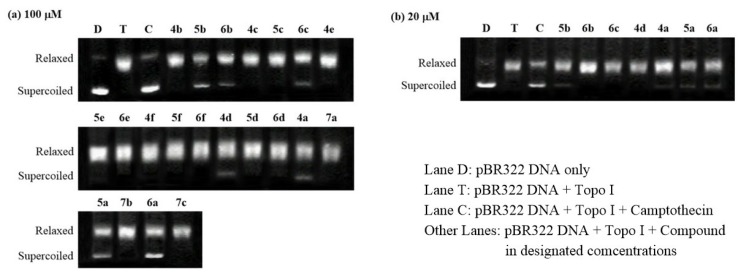
Topoisomerase I inhibitory activity of the prepared compounds **4a**–**f**, **5a**–**f**, **6a**–**f**, and **7a**–**c**. (**a**) The DNA relaxation assay of the prepared compounds at 100 μM; (**b**) The DNA relaxation assay of the selected active compounds at 20 μM.

As shown in Figure 2 and Table 1, compounds **5a**, **5b**, and **6a** displayed moderate and weak inhibitory activities against Topo I at concentrations of 100 μM and 20 μM, respectively. In addition, compounds **4a**, **4b**, **4d**, **6b**, and **6c** also showed mild Topo I inhibitory activities at 100 μM. However, the other compounds, especially compounds with potent anticancer activity such as **5d**, **5f**, and **6f,** were not inhibitory to Topo I even at 100 μM. Therefore, it is evident that the Topo I inhibition of the title compounds only have minor correlations with their anticancer activities.

**Figure 3:**
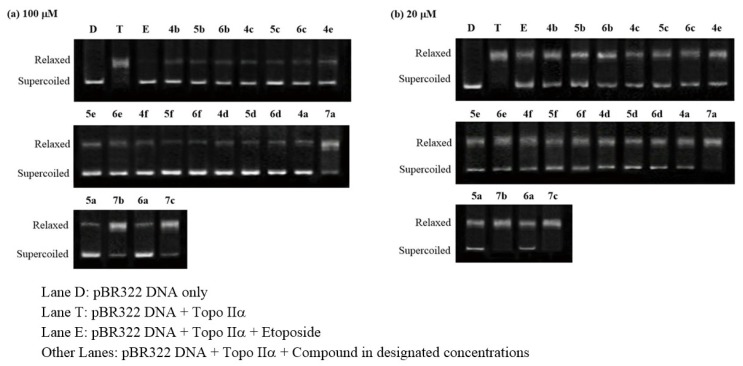
Topoisomerase II inhibitory activity of the prepared compounds **4a**–**f**, **5a**–**f**, **6a**–**f**, and **7a**–**c**. (**a**) The DNA relaxation assay of the prepared compounds at 100 μM; (**b**) The DNA relaxation assay of the prepared compounds at 20 μM.

Concerning Topo II inhibitory activity, it was observed that most compounds exhibited considerable inhibitory activity at concentrations of 100 and 20 μM (Figure 3 and Table 1). Among them, compound **5f** with the strongest cytotoxic activity exhibited the most potent inhibitory activity with inhibition rates of 83.5% and 43.2% at concentrations of 100 and 20 μM, respectively. Compounds **5d** and **6f** also showed significant activities comparable to positive control etoposide. On the contrary, three dimer derivatives **7a**–**c** with poor cytotoxic activities only showed weak inhibitory activities at 100 μM and 20 μM. The close correlations between the Topo II inhibitory activity and cytotoxic activity suggested that Topo II inhibition of the title compounds probably played an important role in their anticancer activity.

### 2.4. Molecular Docking

**Figure 4:**
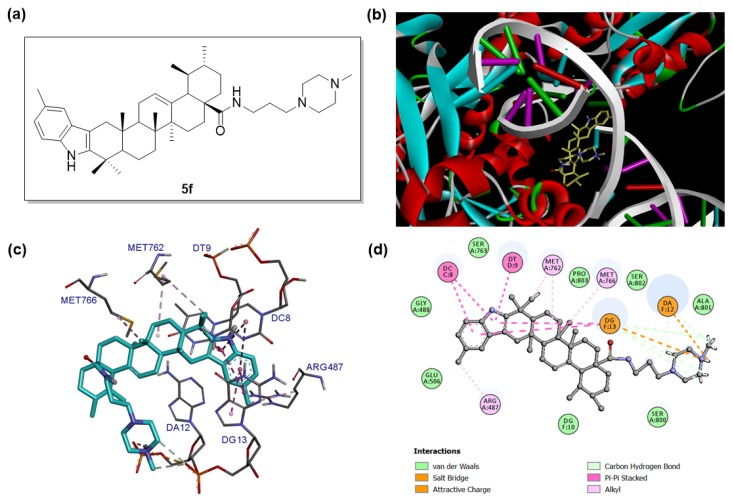
Binding mode of compound **5f** with human topoisomerase IIα in complex with DNA (PDB: 5GWK). (**a**) Molecular structure of compound **5f**. (**b**) Binding pose of compound **5f** within the active site of Topo IIα. Ligand is presented as stick models and colored by atom type, whereas the protein is presented as ribbons and DNA is presented as arrows (for backbone) and ladders (for base pairs). (**c**) Detailed docked view of compound **5f** in the active site. The amino acid residues and base pairs are presented as sticks and colored by atom type, while the interactions are presented as dash lines. (**d**) Two-dimensional projection drawing of compound **5f** docked into the active site of Topo IIα.

To gain more understanding between target compounds and Topo II, the binding modes of compound **5f** with Topo II were explored by molecular docking based on the reported Topo IIα/DNA/inhibitor complex structure (PDB: 5GWK). The docking studies were performed by using the GLIDE docking module of Schrodinger suite 2015-1 and the docking results were analyzed and visualized by Discovery Studio 2016 Client. The binding modes of compound **5f** with Topo IIα/DNA complex are shown in Figure 4.

It was observed that compound **5f** could be suitably docked into the binding site of Topo IIα (Figure 4b,c), affording a significant docking score (−8.240). Specifically, compound **5f** was deeply embedded into the major groove of DNA. The planar 5-methyl-indole moiety of **5f** was intercalated into the gap of DNA double strand and formed π–π stacking interactions with Cytosine DC8, Thymine DT9 and Guanine DG13. The methyl group on the indole ring formed hydrophobic alkyl interactions with Arg487. Other hydrophobic alkyl interactions were also detected between the methyl group (C40), methylene groups (C7 and C8) and MET762, methyl group (C32) and MET766. The *N*-(3-(4-methylpiperazin-1-yl)propyl)carboxamide side chain of compound **5f** also played an important role in the molecular docking. The nitrogen atom (N4) on the piperazine ring formed an electrostatic attractive charge interaction with the phosphate group of guanosine monophosphate residue (DG13), and the hydrogen on N4 formed a salt bridge with the phosphate group of adenosine monophosphate residue (DA12). In addition, compound **5f** also formed van der Waals interactions with residues Gly488, Glu506, Ser763, Pro803, Ser802, Ala801, Ser800, and Guanine DG10 (Figure 4d). Taken together, the molecular docking results in combination with topoisomerase inhibition assay data indicated that compound **5f** could be a promising Topo IIα inhibitor worthy of further investigations.

### 2.5. Annexin V-FITC/PI Staining Assay

**Figure 5:**
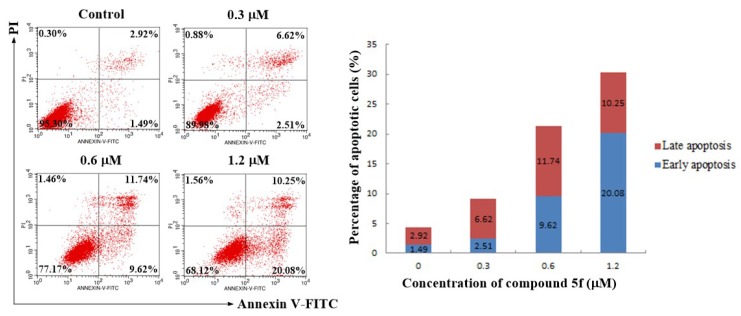
Apoptotic assay by flow cytometry. SMMC-7721 cells were treated with compound **5f** at 0, 0.3, 0.6, and 1.2 μM for 48 h. The cells were stained with Annexin V-FITC/PI and were detected by flow cytometry.

To further investigate the role of apoptosis in the antitumor activity of compound **5f**, SMMC-7721 cells were treated with different concentrations of compound **5f** (0, 0.3, 0.6, and 1.2 μM) for 48 h. Then the treated cells were stained with Annexin V-FITC/PI followed by flow cytometry analysis. As shown in Figure 5, the percentage of early and late apoptotic cells (early apoptosis: Lower right quadrant, AV+/PI−; late apoptosis: Upper right quadrant, AV+/PI+) considerably increased from 4.41% (0 μM) to 9.13% (0.3 μM), 21.36% (0.6 μM), and 30.33% (1.2 μM), respectively. These results indicated that compound **5f** could trigger the apoptosis of SMMC-7721 cells in a dose-dependent manner.

### 2.6. AO/EB Staining Assay

**Figure 6:**
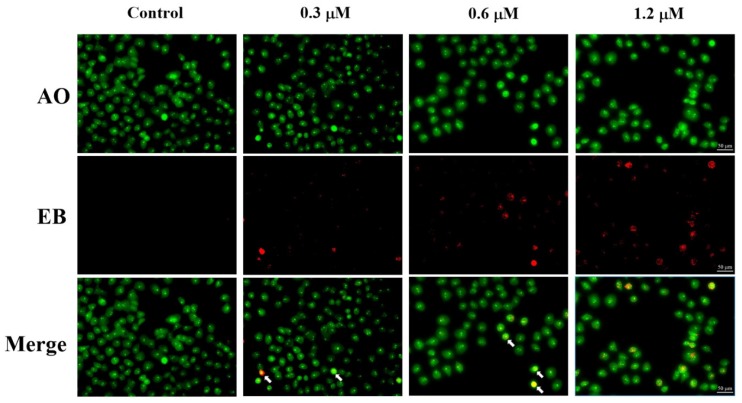
AO/EB staining of compound **5f** in SMMC-7721 cells. SMMC-7721 cells were treated with compound **5f** at 0, 0.3, 0.6, and 1.2 μM for 48 h. The cells were stained with AO/EB and were detected by fluorescence microscope. White arrow: apoptotic cells.

Apoptosis induced by compound **5f** was further evaluated using acridine orange / ethidium bromide (AO/EB) double staining. AO, as a vital dye showing green fluorescence, can pass through cell membrane of living or dead cells, while EB only stains apoptotic or necrotic cells that have lost their integrity and appear red fluorescence. Therefore, the normal cells will be stained only by AO with bright green while apoptotic cells will be stained by AO and EB with red-orange [26,27]. As shown in Figure 6, the cell population in control appeared in green color with usual morphology. Fluorescence images of cells treated with various concentrations of **5f** displayed the increase of cells with red-orange fluorescence, the reduced number of normal cells and also displayed distorted structural characteristics such as chromatin condensation, cell shrinkage and membrane blebbing, which further indicated that compound **5f** induced apoptosis in SMMC-7721 cells.

### 2.7. ROS Generation Assay

**Figure 7:**
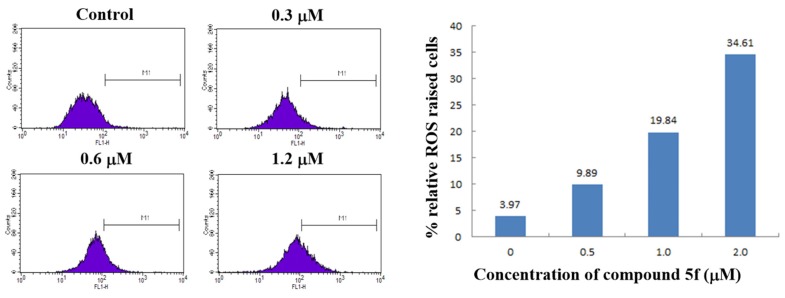
Effects of compound **5f** on the intracellur reactive oxygen species (ROS) level in SMMC-7721 cells. SMMC-7721 cells were treated with different concentrations of compound **5f** (0, 0.3, 0.6, and 1.2 μM) for 48 h, stained with DCFH-DA and analyzed using flow cytometry.

Reactive oxygen species (ROS), which can exert oxidative stress to cells and lead to disturbances in mitochondrial membrane potential, are important mediators for the induction of apoptosis in many cancer cells [28]. To determine whether the apoptosis induced by compound **5f** was associated with intracellular ROS level, SMMC-7721 cells treated with different concentrations of **5f** were stained with the fluorescent probe DCFH-DA and analyzed by flow cytometry. As shown in Figure 7, the cells with elevated ROS levels increased from 3.97% (Control) to 9.89% (0.3 μM), 19.84% (0.6 μM) and 34.61% (1.2 μM), which indicated that compound **5f** could induce ROS generation in SMMC-7721 cells in a dose-dependent manner.

### 2.8. Mitochondrial Membrane Potential Assay

**Figure 8:**
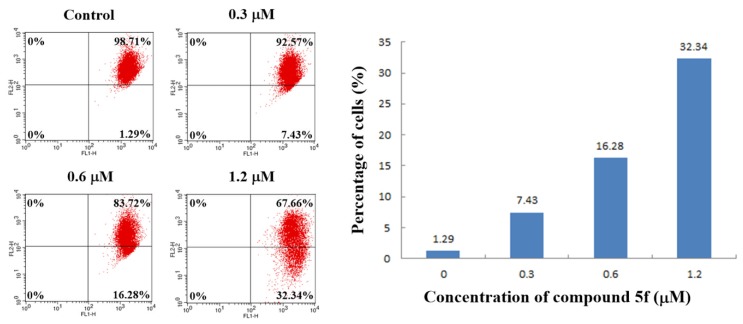
Compound **5f**-induced loss of the mitochondrial membrane potential (ΔΨ_m_). SMMC-7721 cells were treated with compound **5f** (0, 0.3, 0.6, and 1.2 μM) for 24 h, incubated with JC-1, and analyzed using flow cytometry.

It has been widely believed that intracellular ROS accumulation could decrease mitochondrial membrane potential (ΔΨ_m_), which is a characteristic phenomenon of early apoptosis [29]. To investigate the correlation between ΔΨ_m_ and apoptosis induced by compound **5f**, the measurement of cells with decreased ΔΨ_m_ were carried out by JC-1 assay kit on the instructions of the manual. As shown in Figure 8, the percentage of cells with low ΔΨ_m_ (Lower right quadrant) increased from 1.29% (Control) to 7.43% (0.3 μM), 16.28% (0.6 μM), and 32.34% (1.2 μM). Therefore, the observations clearly showed that compound **5f** could decrease mitochondrial membrane potential (ΔΨ_m_) of SMMC-7721 cells in a dose-dependent manner, and the compound probably induced the apoptosis through ROS-mediated mitochondrial pathway.

## 3. Discussion

A series of new indole derivatives of ursolic acid bearing *N*-(aminoalkyl)carboxamide side chains were designed, synthesized, and screened for their in vitro cytotoxic activity against two human hepatocarcinoma cell lines (SMMC-7721, HepG2) and a normal human hepatocyte cell line (LO2). A number of derivatives showed considerable cytotoxic activities in MTT assay. Especially, compound **5f** exhibited the most potent inhibitory activity against SMMC-7721 and HepG2 cells among all derivatives and significantly lower cytotoxicity to LO2 cells. Concerning structure-activity relationships, the order of effects of the *N*-containing moieties to the cytotoxicity could be summarized as *N*-methylpiperazine > NH_2_, N(Me)_2_, N(Et)_2_, and piperidine > morpholine. In addition, 3C linker on the side chain could be more beneficial to the cytotoxicity than 2C and 4C alkyl linkers. In vitro pharmacological studies showed that compound **5f** could significantly inhibit Topoisomerase II activity and thus hinder the relaxation of supercoiled DNA. In addition, it could induce intracellular ROS generation, decrease mitochondrial membrane potential, and finally lead to the apoptosis of SMMC-7721 cells. Due to its strong Topoisomerase II inhibitory activity and anticancer activity, this compound exhibited potential as an anticancer drug candidate. In addition, this class of derivatives could expand the structural diversity of anticancer agents and affirm the perspectives of further studies. More investigations on the structural modification and the structure–activity relationship of this scaffold will be carried out in the future.

## 4. Materials and Methods

### 4.1. General

Melting points were measured on an XT-4 apparatus (Taike Corp., Beijing, China) and were uncorrected. IR spetra were measured on a Nexus 870 FT-IR spectrometer (Thermo Fisher Scientific, Waltham, MA, USA), and the absorption bands were expressed in cm^−1^. The HRMS spectra were recorded on a high-resolution mass spectrometer equipped with electrospray (ESI) and nanospray sources, and a quadrupole-time of flight hybrid analyzer (Q-TOF Premier/nanoAquity, Waters, Milford, MA, USA). ^1^H and ^13^C NMR spectra were obtained in CDCl_3_ on a Bruker DRX-600 NMR spectrometer (Billerica, MA, USA) using TMS as internal standard. Reactions and the resulted products were monitored by TLC which was carried out on TLC Silica gel 60 F_254_ Aluminum sheets from Merck KGaA, Darmstadt, Germany, and visualized in UV light (254 nm). Silica gel (300–400 mesh) for column chromatography was purchased from Qingdao Marine Chemical Factory (Qingdao, China). The reagents (chemicals), all being of A.R. grade, were purchased from Shanghai Chemical Reagent Company (Shanghai, China) and Energy Chemical (Shanghai, China). Ursolic acid (95%) was bought from Jingzhu Biological Technology Co., Ltd. (Nanjing, China).

### 4.2. Procedure for the Synthesis of Compound ***3***

To a solution of ursolic acid (1.0 g, 2.2 mmol) in acetone (150 mL) was added dropwise to 1 mL of Jones reagent (0.27 g CrO_3_ dissolved in 0.23 mL concentrated H_2_SO_4_ and 0.73 mL H_2_O) at 0 °C. The mixture was then stirred at room temperature for 5 h (TLC control). Subsequently, isopropanol (45 mL) was added and the mixture was stirred for additional 0.5 h. Then the mixture was filtered, the filtrate was concentrated in vacuo to remove solvent. The residue was then recrystallized in MeOH to afford compound **2** as a white solid (0.56 g, yield 76%). The spectral data of compound **2** were in accordance with the records of previous literature [30].

To a solution of compound **2** (0.44 g, 1 mmol) in 10 mL EtOH was added 0.33 g (2.1 mmol) of *p*-tolylhydrazine hydrochloride and 0.5 mL concentrated HCl. The mixture was refluxed at 85 °C for 3 h. After cooling, the mixture was poured into 100 mL of ice-cold water and extracted with CH_2_Cl_2_ (3 × 50 mL). The organic layer was combined, washed with water, saturated NaHCO_3_ solution and brine, dried over anhydrous Na_2_SO_4_ and concentrated to give a crude product, which was purified by silica gel column chromatography (petroleum ether–acetone 15:1, *v*/*v*) to afford compound **3** as a white solid (69%, 0.37 g) [25].

### 4.3. General Procedure for the Synthesis of Compounds ***4a**–**f***, ***5a**–**f***, and ***6a**–**f***

To a solution of compound **3** (54 mg, 0.1 mmol) in CH_2_Cl_2_ (5 mL) were added HOBt (19 mg, 0.12 mmol) and DCC (24 mg, 0.12 mmol). The reaction mixture was stirred at room temperature for 0.5 h and different amine (0.12 mmol) was added. The mixture was stirred for 12 h at room temperature. At the end of reaction, the mixture was filtered, and the filtrate concentrated in vacuo. The obtained residue was purified by silica gel column chromatography (petroleum ether–acetone 30:1, *v*/*v*) to afford the corresponding product **4a**–**f**, **5a**–**f**, and **6a**–**f** with 2-, 3- and 4-carbon side chains, respectively (Scheme 1).

#### 4.3.1. *N*-(2-Aminoethyl)-5′-methyl-1′*H*-ursa-2,12-dieno[3,2-*b*]indol-28-carboxamide (**4a**)

Yellow solid; Yield: 72%; mp. 228–230 °C; ^1^H NMR (600 MHz, CDCl_3_): *δ* 0.88 (s, 3H), 0.93 (d, *J* = 6.2 Hz, 3H), 0.95 (s, 3H), 0.96 (d, *J* = 6.5 Hz, 3H), 1.15 (s, 3H), 1.20 (s, 3H), 1.29 (s, 3H), 1.29–1.90 (m, 15H), 1.90–2.08 (m, 2H), 2.08–2.25 (m, 3H), 2.32 (brs, 2H), 2.43 (s, 3H), 2.77 (d, *J* = 14.8 Hz, 1H), 2.83 (brs, 2H), 3.12 (m, 1H), 3.42 (m, 1H), 5.45 (brs, 1H), 6.46 (brs, 1H), 6.93 (d, *J* = 8.0 Hz, 1H), 7.18 (d, *J* = 8.2 Hz, 1H), 7.21 (s, 1H), 7.87 (brs, 1H); ^13^C NMR (150 MHz, CDCl_3_): *δ* 15.92, 16.95, 17.36, 19.39, 21.35, 21.60, 23.35, 23.37, 23.66, 25.01, 28.07, 31.09, 31.12, 32.46, 34.15, 37.23, 37.50, 38.10, 39.19, 39.80, 39.99, 41.19, 41.77, 42.77, 46.38, 48.07, 53.30, 54.10, 106.32, 110.20, 117.81, 122.44, 126.17, 128.10, 128.54, 134.59, 139.51, 141.19, 178.82; IR (KBr, cm^−1^): 3311, 2924, 2854, 1635, 1519, 1457, 1378, 1305, 1276, 794, 737; HRMS (ESI): *m*/*z* [M + H]^+^ calcd. for C_39_H_58_N_3_O: 584.4580; found: 584.4584.

#### 4.3.2. *N*-(2-(Dimethylamino)ethyl)-5′-methyl-1′*H*-ursa-2,12-dieno[3,2-*b*]indol-28-carboxamide (**4b**)

Yellow solid; Yield: 89%; mp. 166–168 °C; ^1^H NMR (600 MHz, CDCl_3_): *δ* 0.90 (s, 3H), 0.93 (d, *J* = 6.4 Hz, 3H), 0.96 (s, 3H), 0.97 (d, *J* = 6.2 Hz, 3H), 1.15 (s, 3H), 1.21 (s, 3H), 1.30 (s, 3H), 1.35–2.00 (m, 16H), 2.10–2.23 (m, 3H), 2.25 (s, 6H), 2.30–2.42 (m, 3H), 2.43 (s, 3H), 2.78 (d, *J* = 14.8 Hz, 1H), 3.19 (m, 1H), 3.35 (m, 1H), 5.41 (brs, 1H), 6.59 (brs, 1H), 6.93 (d, *J* = 8.0 Hz, 1H), 7.18 (d, *J* = 8.2 Hz, 1H), 7.20 (s, 1H), 7.87 (brs, 1H); ^13^C NMR (150 MHz, CDCl_3_): *δ* 15.91, 17.00, 17.34, 19.43, 21.34, 21.59, 23.31, 23.37, 23.69, 25.02, 28.12, 31.08, 31.12, 32.60, 34.18, 36.98, 37.28, 37.41, 38.13, 39.23, 39.86, 40.02, 42.79, 45.35, 46.44, 48.01, 53.40, 54.30, 57.75, 106.33, 110.20, 117.81, 122.41, 126.24, 128.09, 128.59, 134.64, 139.25, 141.22, 178.33; IR (KBr, cm^−1^): 3298, 2923, 2854, 1633, 1510, 1457, 1380, 1307, 1186, 1054, 793; HRMS (ESI): *m*/*z* [M + H]^+^ calcd. for C_41_H_62_N_3_O: 612.4893; found: 612.4887.

#### 4.3.3. *N*-(2-(Diethylamino)ethyl)-5′-methyl-1′*H*-ursa-2,12-dieno[3,2-*b*]indol-28-carboxamide (**4c**)

Yellow solid; Yield: 88%; mp. 163–165 °C; ^1^H NMR (600 MHz, CDCl_3_): *δ* 0.88 (s, 3H), 0.93 (d, *J* = 6.3 Hz, 3H), 0.95 (s, 3H), 0.96 (d, *J* = 6.3 Hz, 3H), 1.04 (t, *J* = 6.9 Hz, 6H), 1.15 (s, 3H), 1.21 (s, 3H), 1.30 (s, 3H), 1.31–2.00 (m, 16H), 2.12 (m, 1H), 2.18–2.25 (m, 2H), 2.43 (s, 3H), 2.53 (m, 4H), 2.56–2.61 (m, 3H), 2.78 (d, *J* = 14.8 Hz, 1H), 3.10 (m, 1H), 3.42 (m, 1H), 5.41 (brs, 1H), 6.61 (brs, 1H), 6.93 (d, *J* = 8.0 Hz, 1H), 7.19 (d, *J* = 8.1 Hz, 1H), 7.21 (s, 1H), 7.96 (brs, 1H); ^13^C NMR (150 MHz, CDCl_3_): *δ* 11.79, 15.82, 16.85, 17.42, 19.40, 21.34, 21.59, 23.34, 23.36, 23.55, 24.96, 28.05, 31.08, 31.09, 32.43, 34.14, 36.87, 37.17, 37.51, 38.10, 39.29, 39.77, 39.95, 42.66, 46.38, 46.64, 47.92, 51.40, 53.32, 54.15, 106.25, 110.19, 117.76, 122.34, 125.99, 128.01, 128.53, 134.59, 139.24, 141.23, 178.03; IR (KBr, cm^−1^): 3393, 3301, 2924, 2854, 1634, 1508, 1457, 1378, 1306, 1185, 1083, 795; HRMS (ESI): *m*/*z* [M + H]^+^ calcd. for C_43_H_66_N_3_O: 640.5206; found: 640.5203.

#### 4.3.4. *N*-(2-(Piperidin-1-yl)ethyl)-5′-methyl-1′*H*-ursa-2,12-dieno[3,2-*b*]indol-28-carboxamide (**4d**)

Yellow solid; Yield: 90%; mp. 195–197 °C; ^1^H NMR (600 MHz, CDCl_3_): *δ* 0.89 (s, 3H), 0.94 (d, *J* = 6.5 Hz, 3H), 0.95 (s, 3H), 0.97 (d, *J* = 6.5 Hz, 3H), 1.16 (s, 3H), 1.21 (s, 3H), 1.30 (s, 3H), 1.35–2.10 (m, 23H), 2.10–2.26 (m, 3H), 2.36–2.42 (m, 6H), 2.43 (s, 3H), 2.78 (d, *J* = 14.8 Hz, 1H), 3.23 (m, 1H), 3.37 (m, 1H), 5.44 (brs, 1H), 6.62 (brs, 1H), 6.93 (d, *J* = 8.0 Hz, 1H), 7.18 (d, *J* = 8.2 Hz, 1H), 7.21 (s, 1H), 7.85 (brs, 1H); ^13^C NMR (150 MHz, CDCl_3_): *δ* 15.87, 16.90, 17.45, 19.44, 21.36, 21.59, 23.35, 23.37, 23.63, 24.48, 25.02, 26.18, 28.09, 31.13, 31.15, 32.48, 34.18, 36.08, 37.24, 37.51, 38.15, 39.27, 39.84, 40.03, 42.75, 46.43, 48.00, 53.37, 54.25, 54.44, 57.15, 106.34, 110.20, 117.79, 122.42, 126.04, 128.09, 128.58, 134.64, 139.29, 141.22, 178.08; IR (KBr, cm^−1^): 3312, 2926, 2853, 1633, 1508, 1456, 1379, 1305, 1127, 794, 736; HRMS (ESI): *m*/*z* [M + H]^+^ calcd. for C_44_H_66_N_3_O: 652.5206; found: 652.5212.

#### 4.3.5. *N*-(2-Morpholinoethyl)-5′-methyl-1′*H*-ursa-2,12-dieno[3,2-*b*]indol-28-carboxamide (**4e**)

Yellow solid; Yield: 82%; mp. 104–105 °C; ^1^H NMR (600 MHz, CDCl_3_): *δ* 0.87 (s, 3H), 0.93 (d, *J* = 6.5 Hz, 3H), 0.94 (s, 3H), 0.98 (d, *J* = 6.5 Hz, 3H), 1.16 (s, 3H), 1.20 (s, 3H), 1.34 (s, 3H), 1.36–2.35 (m, 22H), 2.43 (s, 3H), 2.50 (brs, 4H), 2.77 (d, *J* = 14.8 Hz, 1H), 3.26 (m, 1H), 3.43 (m, 1H), 3.76 (brs, 4H), 5.44 (brs, 1H), 6.53 (brs, 1H), 6.93 (d, *J* = 8.0 Hz, 1H), 7.18 (d, *J* = 8.1 Hz, 1H), 7.21 (s, 1H), 7.76 (brs, 1H); ^13^C NMR (150 MHz, CDCl_3_): *δ* 15.92, 16.97, 17.45, 19.44, 21.36, 21.60, 22.82, 23.41, 23.69, 25.05, 25.79, 28.10, 30.36, 31.16, 31.58, 34.18, 37.24, 37.53, 38.16, 39.30, 39.85, 40.05, 42.83, 46.40, 48.10, 53.36, 53.54, 54.32, 57.14, 67.01, 106.37, 110.19, 117.87, 122.51, 125.89, 128.21, 128.59, 134.64, 139.63, 141.14, 178.22; IR (KBr, cm^−1^): 3313, 2954, 2923, 2852, 1635, 1498, 1458, 1379, 1303, 1266, 1187, 1119, 1081, 967, 792, 739; HRMS (ESI): *m*/*z* [M + H]^+^ calcd. for C_43_H_64_N_3_O_2_: 654.4999; found: 654.4995.

#### 4.3.6. *N*-(2-(4-Methylpiperazin-1-yl)ethyl)-5′-methyl-1′*H*-ursa-2,12-dieno[3,2-*b*]indol-28-carboxamide (**4f**)

Yellow solid; Yield: 87%; mp. 188–190 °C; ^1^H NMR (600 MHz, CDCl_3_): *δ* 0.87 (s, 3H), 0.94 (d, *J* = 6.5 Hz, 3H), 0.95 (s, 3H), 0.97 (d, *J* = 6.4 Hz, 3H), 1.15 (s, 3H), 1.20 (s, 3H), 1.29 (s, 3H), 1.31–2.05 (m, 16H), 2.10–2.25 (m, 3H), 2.31 (s, 3H), 2.43 (s, 3H), 2.48–2.60 (m, 12H), 2.77 (d, *J* = 14.8 Hz, 1H), 3.23 (m, 1H), 3.37 (m, 1H), 5.43 (brs, 1H), 6.53 (brs, 1H), 6.92 (d, *J* = 8.2 Hz, 1H), 7.18 (d, *J* = 8.1 Hz, 1H), 7.21 (s, 1H), 7.97 (brs, 1H); ^13^C NMR (150 MHz, CDCl_3_): *δ* 15.96, 16.87, 17.43, 19.37, 21.34, 21.58, 23.27, 23.32, 23.66, 25.01, 28.01, 31.08, 31.09, 32.39, 34.13, 36.00, 37.22, 37.43, 38.09, 39.22, 39.75, 39.98, 42.75, 46.13, 46.34, 47.99, 52.88, 53.27, 54.21, 55.28, 56.46, 106.17, 110.21, 117.70, 122.37, 125.91, 128.01, 128.46, 134.58, 139.44, 141.20, 178.16; IR (KBr, cm^−1^): 3404, 3311, 2924, 2868, 2853, 2791, 1635, 1507, 1457, 1377, 1301, 1165, 1094, 1013, 795, 736; HRMS (ESI): *m*/*z* [M + H]^+^ calcd. for C_44_H_67_N_4_O: 667.5315; found: 667.5309.

#### 4.3.7. *N*-(3-Aminopropyl)-5′-methyl-1′*H*-ursa-2,12-dieno[3,2-*b*]indol-28-carboxamide (**5a**)

Yellow solid; Yield: 73%; mp. 205–207 °C; ^1^H NMR (600 MHz, CDCl_3_): *δ* 0.87 (s, 3H), 0.91 (d, *J* = 6.4 Hz, 3H), 0.95 (s, 3H), 0.96 (d, *J* = 6.1 Hz, 3H), 1.14 (s, 3H), 1.20 (s, 3H), 1.29 (s, 3H), 1.32–2.10 (m, 19H), 2.10–2.30 (m, 3H), 2.42 (s, 3H), 2.62 (brs, 2H), 2.76 (d, *J* = 14.6 Hz, 1H), 2.78 (m, 2H), 3.13 (m, 1H), 3.44 (m, 1H), 5.42 (brs, 1H), 6.39 (brs, 1H), 6.93 (d, *J* = 8.2 Hz, 1H), 7.18 (d, *J* = 8.2 Hz, 1H), 7.20 (s, 1H), 7.82 (brs, 1H); ^13^C NMR (150 MHz, CDCl_3_): *δ* 15.91, 16.91, 17.39, 19.40, 21.35, 21.60, 23.36, 23.39, 23.65, 25.02, 26.67, 28.04, 31.08, 31.14, 32.42, 34.16, 37.22, 37.56, 38.12, 39.23, 39.65, 39.79, 40.00, 42.79, 46.38, 47.99, 53.30, 54.07, 55.34, 106.34, 110.19, 117.82, 122.46, 126.05, 128.15, 128.55, 134.59, 139.73, 141.19, 178.87; IR (KBr, cm^−1^): 3294, 2924, 2854, 1637, 1524, 1457, 1379, 1306, 1187, 1078, 963, 794, 738; HRMS (ESI): *m*/*z* [M + H]^+^ calcd. for C_40_H_60_N_3_O: 598.4736; found: 598.4730.

#### 4.3.8. *N*-(3-(Dimethylamino)propyl)-5′-methyl-1′*H*-ursa-2,12-dieno[3,2-*b*]indol-28-carboxamide (**5b**)

Yellow powder solid; Yield: 81%; mp. 186–188 °C; ^1^H NMR (600 MHz, CDCl_3_): *δ* 0.88 (s, 3H), 0.92 (d, *J* = 6.4 Hz, 3H), 0.95 (s, 3H), 0.96 (d, *J* = 7.4 Hz, 3H), 1.15 (s, 3H), 1.21 (s, 3H), 1.30 (s, 3H), 1.35–2.00 (m, 18H), 2.10–2.25 (m, 3H), 2.26 (s, 6H), 2.30–2.40 (m, 3H), 2.43 (s, 3H), 2.77 (d, *J* = 14.8 Hz, 1H), 3.07 (m, 1H), 3.48 (m, 1H), 5.39 (t, *J* = 3.2 Hz, 1H), 6.93 (d, *J* = 8.2 Hz, 1H), 6.96 (brs, 1H), 7.18 (d, *J* = 8.2 Hz, 1H), 7.20 (s, 1H), 7.81 (brs, 1H); ^13^C NMR (150 MHz, CDCl_3_): *δ* 15.86, 16.94, 17.49, 19.44, 21.36, 21.59, 23.38, 23.49, 23.61, 24.92, 26.35, 28.08, 31.06, 31.14, 32.51, 34.17, 37.22, 37.70, 38.15, 39.21, 39.39, 39.83, 39.94, 42.69, 45.68, 46.44, 47.82, 53.38, 54.08, 58.76, 106.41, 110.19, 117.83, 122.44, 125.91, 128.13, 128.60, 134.65, 139.45, 141.24, 177.93; IR (KBr, cm^−1^): 3411, 3298, 2946, 2924, 2866, 1636, 1522, 1459, 1380, 1305, 1185, 1050, 794; HRMS (ESI): *m*/*z* [M + H]^+^ calcd. for C_42_H_6__4_N_3_O: 626.5049; found: 626.5053.

#### 4.3.9. *N*-(3-(Diethylamino)propyl)-5′-methyl-1′*H*-ursa-2,12-dieno[3,2-*b*]indol-28-carboxamide (**5c**)

Yellow solid; Yield: 87%; mp. 147–149 °C; ^1^H NMR (600 MHz, CDCl_3_): *δ* 0.88 (s, 3H), 0.93 (d, *J* = 6.4 Hz, 3H), 0.96 (s, 3H), 0.97 (d, *J* = 6.5 Hz, 3H), 1.06 (t, *J* = 7.1 Hz, 6H), 1.15 (s, 3H), 1.21 (s, 3H), 1.30 (s, 3H), 1.39–1.85 (m, 16H), 1.95–2.05 (m, 3H), 2.13 (m, 1H), 2.15–2.25 (m, 2H), 2.43 (s, 3H), 2.51 (t, *J* = 6.4 Hz, 2H), 2.58 (q, *J* = 7.1 Hz, 4H), 2.77 (d, *J* = 14.8 Hz, 1H), 3.10 (m, 1H), 3.42 (m, 1H), 5.41 (brs, 1H), 6.65 (brs, 1H), 6.93 (d, *J* = 8.1 Hz, 1H), 7.18 (d, *J* = 8.1 Hz, 1H), 7.21 (s, 1H), 7.85 (brs, 1H); ^13^C NMR (150 MHz, CDCl_3_): *δ* 11.28, 15.85, 16.95, 17.39, 19.44, 21.37, 21.59, 23.37, 23.46, 23.61, 24.92, 26.29, 28.10, 31.11, 31.13, 32.51, 34.17, 37.22, 37.60, 38.15, 39.00, 39.25, 39.82, 39.93, 42.70, 46.44, 46.86, 47.78, 51.54, 53.38, 53.97, 106.36, 110.20, 117.81, 122.41, 125.93, 128.09, 128.58, 134.64, 139.56, 141.21, 177.97; IR (KBr, cm^−1^): 3411, 3304, 2962, 2925, 2855, 1636, 1520, 1456, 1380, 1306, 1188, 1084, 794, 737; HRMS (ESI): *m*/*z* [M + H]^+^ calcd. for C_44_H_68_N_3_O: 654.5362; found: 654.5358.

#### 4.3.10. *N*-(3-(Piperidin-1-yl)propyl)-5′-methyl-1′*H*-ursa-2,12-dieno[3,2-*b*]indol-28-carboxamide (**5d**)

Yellow solid; Yield: 88%; mp. 183–185 °C; ^1^H NMR (600 MHz, CDCl_3_): *δ* 0.87 (s, 3H), 0.93 (d, *J* = 6.4 Hz, 3H), 0.95 (s, 3H), 0.96 (d, *J* = 6.5 Hz, 3H), 1.15 (s, 3H), 1.21 (s, 3H), 1.29 (s, 3H), 1.30–1.90 (m, 23H), 1.90–2.06 (m, 2H), 2.07–2.25 (m, 3H), 2.30–2.40 (m, 6H), 2.43 (s, 3H), 2.77 (d, *J* = 14.8 Hz, 1H), 3.10 (m, 1H), 3.41 (m, 1H), 5.42 (brs, 1H), 6.66 (brs, 1H), 6.93 (d, *J* = 8.0 Hz, 1H), 7.18 (d, *J* = 8.1 Hz, 1H), 7.20 (s, 1H), 7.82 (brs, 1H); ^13^C NMR (150 MHz, CDCl_3_): *δ* 15.87, 17.01, 17.38, 19.45, 21.38, 21.59, 23.39, 23.52, 23.67, 24.37, 24.87, 25.64, 25.88, 28.07, 31.09, 31.15, 32.54, 34.18, 37.23, 37.62, 38.16, 39.12, 39.23, 39.84, 39.92, 42.69, 45.40, 46.45, 47.78, 53.38, 53.97, 54.87, 106.41, 110.19, 117.83, 122.44, 125.90, 128.13, 128.60, 134.64, 139.58, 141.21, 178.01; IR (KBr, cm^−1^): 3300, 2925, 2854, 1635, 1522, 1455, 1379, 1306, 1188, 1125, 794, 737; HRMS (ESI): *m*/*z* [M + H]^+^ calcd. for C_45_H_68_N_3_O: 666.5362; found: 666.5369.

#### 4.3.11. *N*-(3-Morpholinopropyl)-5′-methyl-1′*H*-ursa-2,12-dieno[3,2-*b*]indol-28-carboxamide (**5e**)

Yellow solid; Yield: 90%; mp. 108–110 °C; ^1^H NMR (600 MHz, CDCl_3_): *δ* 0.87 (s, 3H), 0.93 (d, *J* = 6.3 Hz, 3H), 0.95 (s, 3H), 0.96 (d, *J* = 6.5 Hz, 3H), 1.15 (s, 3H), 1.20 (s, 3H), 1.33 (s, 3H), 1.36–2.35 (m, 22H), 2.42 (s, 3H), 2.43–2.50 (m, 2H), 2.51 (brs, 4H), 2.77 (d, *J* = 14.8 Hz, 1H), 3.09 (m, 1H), 3.44 (m, 1H), 3.77 (brs, 4H), 5.41 (brs, 1H), 6.45 (brs, 1H), 6.93 (d, *J* = 8.0 Hz, 1H), 7.18 (d, *J* = 8.1 Hz, 1H), 7.20 (s, 1H), 7.89 (brs, 1H); ^13^C NMR (150 MHz, CDCl_3_): *δ* 15.87, 16.97, 17.39, 19.40, 21.35, 21.59, 23.37, 23.43, 23.65, 24.93, 25.47, 28.03, 31.04, 31.11, 32.46, 34.15, 37.20, 37.58, 38.11, 38.49, 39.25, 39.78, 39.93, 42.73, 46.37, 47.85, 53.30, 53.79, 54.08, 57.37, 66.71, 106.27, 110.19, 117.79, 122.41, 125.89, 128.09, 128.51, 134.59, 139.72, 141.17, 178.21; IR (KBr, cm^−1^): 3313, 2954, 2923, 2853, 1638, 1520, 1459, 1378, 1305, 1186, 1119, 1082, 968, 795, 739; HRMS (ESI): *m*/*z* [M + H]^+^ calcd. for C_44_H_66_N_3_O_2_: 668.5155; found: 668.5161.

#### 4.3.12. *N*-(3-(4-Methylpiperazin-1-yl)propyl)-5′-methyl-1′*H*-ursa-2,12-dieno[3,2-*b*]indol-28-carboxamide (**5f**)

Yellow solid; Yield: 88%; mp. 171–173 °C; ^1^H NMR (600 MHz, CDCl_3_): *δ* 0.86 (s, 3H), 0.93 (d, *J* = 6.4 Hz, 3H), 0.95 (s, 3H), 0.96 (d, *J* = 6.4 Hz, 3H), 1.14 (s, 3H), 1.20 (s, 3H), 1.29 (s, 3H), 1.30–1.90 (m, 17H), 1.95–2.25 (m, 5H), 2.30 (s, 3H), 2.40 (m, 4H), 2.42 (s, 3H), 2.42–2.52 (m, 6H), 2.77 (d, *J* = 14.8 Hz, 1H), 3.07 (m, 1H), 3.45 (m, 1H), 5.41 (brs, 1H), 6.51 (brs, 1H), 6.92 (d, *J* = 8.0 Hz, 1H), 7.17 (d, *J* = 8.1 Hz, 1H), 7.20 (s, 1H), 7.95 (brs, 1H); ^13^C NMR (150 MHz, CDCl_3_): *δ* 15.85, 16.92, 17.37, 19.38, 21.32, 21.58, 23.34, 23.44, 23.63, 24.86, 25.77, 28.01, 31.03, 31.09, 32.44, 34.13, 37.18, 37.53, 38.09, 38.89, 39.21, 39.76, 39.91, 42.68, 46.04, 46.36, 47.78, 53.29, 53.36, 54.02, 55.07, 57.24, 106.23, 110.18, 117.75, 122.36, 125.86, 128.02, 128.49, 134.58, 139.68, 141.19, 178.02; IR (KBr, cm^−1^): 3295, 2924, 2870, 2847, 2796, 1637, 1520, 1458, 1376, 1303, 1283, 1186, 1014, 794, 737; HRMS (ESI): *m*/*z* [M + H]^+^ calcd. for C_45_H_69_N_4_O: 681.5471; found: 681.5475.

#### 4.3.13. *N*-(4-Aminobutyl)-5′-methyl-1′*H*-ursa-2,12-dieno[3,2-*b*]indol-28-carboxamide (**6a**)

Yellow solid; Yield: 72%; mp. 256–258 °C; ^1^H NMR (600 MHz, CDCl_3_): *δ* 0.87 (s, 3H), 0.92 (d, *J* = 6.4 Hz, 3H), 0.95 (s, 3H), 0.96 (d, *J* = 6.4 Hz, 3H), 1.15 (s, 3H), 1.20 (s, 3H), 1.29 (s, 3H), 1.30–2.10 (m, 21H), 2.10–2.30 (m, 3H), 2.43 (s, 3H), 2.45 (brs, 2H), 2.76 (t, *J* = 5.2 Hz, 2H), 2.77 (d, *J* = 15.2 Hz, 1H), 3.03 (m, 1H), 3.36 (m, 1H), 5.43 (brs, 1H), 6.09 (t, *J* = 5.3 Hz, 1H), 6.93 (dd, *J* = 8.2, 1.1 Hz, 1H), 7.18 (d, *J* = 8.2 Hz, 1H), 7.20 (s, 1H), 7.86 (brs, 1H); ^13^C NMR (150 MHz, CDCl_3_): *δ* 15.93, 16.93, 17.39, 19.39, 21.36, 21.61, 23.31, 23.39, 23.66, 25.08, 26.87, 28.05, 31.09, 31.13, 31.56, 32.44, 34.16, 37.23, 37.40, 38.12, 39.23, 39.37, 39.78, 40.03, 41.52, 42.84, 46.38, 47.95, 53.31, 54.21, 106.30, 110.19, 117.80, 122.44, 125.98, 128.11, 128.53, 134.58, 139.96, 141.18, 178.41; IR (KBr, cm^−1^): 3309, 2924, 2855, 1638, 1525, 1456, 1381, 1305, 1185, 1078, 967, 792, 738; HRMS (ESI): *m*/*z* [M + H]^+^ calcd. for C_41_H_62_N_3_O: 612.4893; found: 612.4887.

#### 4.3.14. *N*-(4-(Dimethylamino)butyl)-5′-methyl-1′*H*-ursa-2,12-dieno[3,2-*b*]indol-28-carboxamide (**6b**)

Yellow solid; Yield: 90%; mp. 163–165 °C; ^1^H NMR (600 MHz, CDCl_3_): *δ* 0.86 (s, 3H), 0.91 (d, *J* = 6.4 Hz, 3H), 0.95 (s, 3H), 0.96 (d, *J* = 6.0 Hz, 3H), 1.14 (s, 3H), 1.20 (s, 3H), 1.29 (s, 3H), 1.35–2.00 (m, 20H), 2.10–2.25 (m, 3H), 2.32 (s, 6H), 2.30–2.42 (m, 3H), 2.43 (s, 3H), 2.77 (d, *J* = 14.8 Hz, 1H), 3.03 (m, 1H), 3.35 (m, 1H), 5.41 (t, *J* = 3.1 Hz, 1H), 6.15 (t, *J* = 5.3 Hz, 1H), 6.92 (d, *J* = 8.1 Hz, 1H), 7.18 (d, *J* = 8.2 Hz, 1H), 7.20 (s, 1H), 7.99 (brs, 1H); ^13^C NMR (150 MHz, CDCl_3_): *δ* 15.89, 16.87, 17.36, 19.38, 21.34, 21.60, 23.32, 23.35, 23.63, 24.49, 24.99, 27.15, 28.02, 31.05, 31.11, 32.41, 34.15, 37.21, 37.42, 38.09, 39.16, 39.20, 39.76, 39.98, 42.78, 44.93, 46.36, 47.89, 53.30, 54.09, 58.86, 106.22, 110.22, 117.76, 122.38, 125.95, 128.04, 128.50, 134.59, 139.84, 141.22, 178.35; IR (KBr, cm^−1^): 3287, 2924, 2855, 1637, 1523, 1459, 1306, 1276, 1187, 1083, 794, 737; HRMS (ESI): *m*/*z* [M + H]^+^ calcd. for C_43_H_66_N_3_O: 640.5206; found: 640.5201.

#### 4.3.15. *N*-(4-(Diethylamino)butyl)-5′-methyl-1′*H*-ursa-2,12-dieno[3,2-*b*]indol-28-carboxamide (**6c**)

Yellow solid; Yield: 88%; mp. 132–134 °C; ^1^H NMR (600 MHz, CDCl_3_): *δ* 0.88 (s, 3H), 0.92 (d, *J* = 6.4 Hz, 3H), 0.96 (s, 3H), 0.97 (d, *J* = 6.4 Hz, 3H), 1.02 (t, *J* = 7.1 Hz, 6H), 1.15 (s, 3H), 1.21 (s, 3H), 1.30 (s, 3H), 1.36–2.05 (m, 21H), 2.13 (m, 1H), 2.18–2.25 (m, 2H), 2.43 (s, 3H), 2.45 (m, 2H), 2.53 (q, *J* = 7.1 Hz, 4H), 2.77 (d, *J* = 14.8 Hz, 1H), 3.04 (m, 1H), 3.37 (m, 1H), 5.43 (brs, 1H), 6.01 (brs, 1H), 6.93 (d, *J* = 8.2 Hz, 1H), 7.18 (d, *J* = 8.2 Hz, 1H), 7.21 (s, 1H), 7.88 (brs, 1H); ^13^C NMR (150 MHz, CDCl_3_): *δ* 11.61, 15.90, 16.91, 17.38, 19.40, 21.34, 21.58, 23.31, 23.34, 23.66, 24.73, 25.08, 27.54, 28.07, 31.09, 31.11, 32.45, 34.17, 37.26, 37.37, 38.12, 39.26, 39.53, 39.80, 40.04, 42.86, 46.41, 46.93, 47.94, 52.60, 53.35, 54.27, 106.26, 110.21, 117.77, 122.41, 125.97, 128.07, 128.54, 134.64, 140.03, 141.21, 178.22; IR (KBr, cm^−1^): 3304, 2964, 2926, 2869, 1637, 1524, 1456, 1379, 1306, 1262, 1086, 1051, 796; HRMS (ESI): *m*/*z* [M + H]^+^ calcd. for C_45_H_70_N_3_O: 668.5519; found: 668.5523.

#### 4.3.16. *N*-(4-(Piperidin-1-yl)butyl)-5′-methyl-1′*H*-ursa-2,12-dieno[3,2-*b*]indol-28-carboxamide (**6d**)

Yellow solid; Yield: 90%; mp. 121–123 °C; ^1^H NMR (600 MHz, CDCl_3_): *δ* 0.88 (s, 3H), 0.93 (d, *J* = 6.4 Hz, 3H), 0.96 (s, 3H), 0.97 (d, *J* = 6.5 Hz, 3H), 1.15 (s, 3H), 1.21 (s, 3H), 1.30 (s, 3H), 1.37–2.08 (m, 27H), 2.08–2.25 (m, 3H), 2.25–2.42 (m, 6H), 2.43 (s, 3H), 2.77 (d, *J* = 14.8 Hz, 1H), 3.03 (m, 1H), 3.35 (m, 1H), 5.43 (brs, 1H), 6.02 (brs, 1H), 6.93 (d, *J* = 8.1 Hz, 1H), 7.18 (d, *J* = 8.1 Hz, 1H), 7.21 (s, 1H), 7.85 (brs, 1H); ^13^C NMR (150 MHz, CDCl_3_): *δ* 15.91, 16.93, 17.38, 19.41, 21.35, 21.59, 23.33, 23.37, 23.67, 24.18, 24.30, 25.08, 25.64, 27.51, 28.08, 31.11, 31.14, 32.47, 34.17, 37.27, 37.41, 38.13, 39.25, 39.45, 39.82, 40.04, 42.86, 46.43, 47.94, 53.35, 54.22, 54.46, 58.76, 106.34, 110.21, 117.81, 122.46, 125.99, 128.12, 128.57, 134.65, 140.01, 141.22, 178.28; IR (KBr, cm^−1^): 3298, 2924, 2853, 1637, 1526, 1456, 1379, 1306, 1188, 1082, 963, 793, 738; HRMS (ESI): *m*/*z* [M + H]^+^ calcd. for C_46_H_70_N_3_O: 680.5519; found: 680.5516.

#### 4.3.17. *N*-(4-Morpholinobutyl)-5′-methyl-1′*H*-ursa-2,12-dieno[3,2-*b*]indol-28-carboxamide (**6e**)

Yellow solid; Yield: 86%; mp. 221–223 °C; ^1^H NMR (600 MHz, CDCl_3_): *δ* 0.88 (s, 3H), 0.92 (d, *J* = 6.4 Hz, 3H), 0.96 (s, 3H), 0.97 (d, *J* = 6.5 Hz, 3H), 1.15 (s, 3H), 1.21 (s, 3H), 1.29 (s, 3H), 1.30–2.05 (m, 23H), 2.13 (m, 1H), 2.22 (m, 2H), 2.35 (brs, 4H), 2.43 (s, 3H), 2.77 (d, *J* = 14.8 Hz, 1H), 3.04 (m, 1H), 3.37 (m, 1H), 3.71 (m, 4H), 5.43 (brs, 1H), 5.99 (t, *J* = 5.5 Hz, 1H), 6.93 (d, *J* = 8.2 Hz, 1H), 7.18 (d, *J* = 8.0 Hz, 1H), 7.21 (s, 1H), 7.93 (brs, 1H); ^13^C NMR (150 MHz, CDCl_3_): *δ* 15.91, 16.90, 17.39, 19.37, 21.34, 21.59, 23.29, 23.35, 23.64, 24.18, 25.08, 27.39, 28.04, 31.07, 31.10, 32.42, 34.14, 37.23, 37.37, 38.09, 39.26, 39.48, 39.77, 40.01, 42.85, 46.37, 47.93, 53.28, 53.81, 54.27, 58.65, 67.01, 106.22, 110.20, 117.78, 122.42, 125.93, 128.07, 128.50, 134.60, 140.08, 141.17, 178.27; IR (KBr, cm^−1^): 3314, 2953, 2925, 2853, 1635, 1530, 1456, 1376, 1305, 1274, 1118, 1084, 788, 732; HRMS (ESI): *m*/*z* [M + H]^+^ calcd. for C_45_H_68_N_3_O_2_: 682.5312; found: 682.5317.

#### 4.3.18. *N*-(4-(4-Methylpiperazin-1-yl)butyl)-5′-methyl-1′*H*-ursa-2,12-dieno[3,2-*b*]indol-28-carboxamide (**6f**)

Yellow solid; Yield: 91%; mp. 146–148 °C; ^1^H NMR (600 MHz, CDCl_3_): *δ* 0.87 (s, 3H), 0.92 (d, *J* = 6.1 Hz, 3H), 0.96 (s, 3H), 0.97 (d, *J* = 6.5 Hz, 3H), 1.15 (s, 3H), 1.21 (s, 3H), 1.29 (s, 3H), 1.30–2.10 (m, 20H), 2.10–2.25 (m, 3H), 2.28 (s, 3H), 2.35 (brs, 4H), 2.43 (s, 3H), 2.44–2.51 (m, 7H), 2.77 (d, *J* = 14.8 Hz, 1H), 3.04 (m, 1H), 3.35 (m, 1H), 5.42 (brs, 1H), 5.99 (brs, 1H), 6.93 (d, *J* = 8.0 Hz, 1H), 7.18 (d, *J* = 8.0 Hz, 1H), 7.20 (s, 1H), 7.88 (brs, 1H); ^13^C NMR (150 MHz, CDCl_3_): *δ* 15.94, 16.91, 17.38, 19.39, 21.33, 21.57, 23.30, 23.35, 23.66, 24.51, 25.08, 27.43, 28.06, 31.08, 31.11, 32.44, 34.16, 37.26, 37.37, 38.11, 39.25, 39.53, 39.80, 40.03, 42.86, 46.01, 46.40, 47.94, 53.13, 53.33, 54.27, 55.07, 58.16, 106.27, 110.20, 117.78, 122.42, 125.95, 128.08, 128.54, 134.62, 140.04, 141.19, 178.26; IR (KBr, cm^−1^): 3289, 2925, 2854, 2799, 1638, 1522, 1457, 1375, 1305, 1282, 1165, 1013, 794, 737; HRMS (ESI): *m*/*z* [M + H]^+^ calcd. for C_46_H_71_N_4_O: 695.5628; found: 695.5620.

### 4.4. General Procedure for the Synthesis of Compounds ***7a**–**c***

To a solution of compound **3** (98 mg, 0.18 mmol) in CH_2_Cl_2_ (5 mL) were added HOBt (36 mg, 0.24 mmol) and DCC (48 mg, 0.24 mmol). The reaction mixture was stirred at room temperature for 0.5 h and then ethane-1,2-diamine, propane-1,3-diamine, or butane-1,4-diamine (0.09 mmol) was added. The mixture was stirred for 12 h at room temperature. At the end of reaction, the mixture was worked up as above to afford the corresponding dimer product **7a**–**c** with 2-, 3- and 4-carbon chain, respectively (Scheme 2).

#### 4.4.1. *N*,*N*′-(ethane-1,2-diyl)-bis(5′-methyl-1′*H*-ursa-2,12-dieno[3,2-*b*]indol-28-carboxamide) (**7a**)

Yellow solid; Yield: 61%; mp. 301–303 °C; ^1^H NMR (600 MHz, CDCl_3_): *δ* 0.86 (s, 6H), 0.93 (d, *J* = 6.2 Hz, 6H), 0.95 (s, 6H), 0.98 (s, 6H), 1.16 (s, 6H), 1.19 (s, 6H), 1.29 (s, 6H), 1.30–2.10 (m, 34H), 2.10–2.28 (m, 6H), 2.44 (s, 6H), 2.77 (d, *J* = 14.8 Hz, 2H), 3.14 (m, 2H), 3.48 (m, 2H), 5.48 (brs, 2H), 6.58 (brs, 2H), 6.93 (d, *J* = 7.9 Hz, 2H), 7.18 (d, *J* = 8.0 Hz, 2H), 7.21 (s, 2H), 7.86 (brs, 1H), 7.92 (brs, 1H); ^13^C NMR (150 MHz, CDCl_3_): *δ* 15.88, 16.85, 17.39, 19.38, 21.39, 21.61, 23.35, 23.39, 23.64, 25.02, 25.07, 28.08, 31.11, 32.37, 34.07, 34.14, 37.19, 37.65, 38.11, 39.22, 39.77, 39.97, 42.68, 46.38, 47.86, 53.30, 53.73, 106.30, 110.18, 117.84, 122.42, 126.33, 128.10, 128.54, 134.60, 139.29, 141.20, 179.39; IR (KBr, cm^−1^): 3415, 3322, 2923, 2853, 1639, 1522, 1456, 1379, 1304, 1237, 1083, 963, 792, 732; HRMS (ESI): *m*/*z* [M + H]^+^ calcd. for C_76_H_107_N_4_O_2_: 1107.8394; found: 1107.8387.

#### 4.4.2. *N*,*N*′-(propane-1,3-diyl)-bis(5′-methyl-1′*H*-ursa-2,12-dieno[3,2-*b*]indol-28-carboxamide) (**7b**)

Yellow solid; Yield: 65%; mp. 276–278 °C; ^1^H NMR (600 MHz, CDCl_3_): *δ* 0.87 (s, 6H), 0.92–0.98 (m, 18H), 1.15 (s, 6H), 1.16 (s, 3H), 1.21 (s, 3H), 1.28 (s, 3H), 1.33 (s, 3H), 1.30–2.10 (m, 36H), 2.10–2.26 (m, 6H), 2.42 (s, 3H), 2.43 (s, 3H), 2.77 (m, 2H), 3.13 (m, 2H), 3.27 (m, 2H), 5.37 (brs, 1H), 5.48 (brs, 1H), 6.92 (d, *J* = 7.1 Hz, 1H), 6.93 (d, *J* = 7.1 Hz, 1H), 7.17 (d, *J* = 7.7 Hz, 1H), 7.18 (d, *J* = 7.7 Hz, 1H), 7.20 (s, 2H), 7.93 (brs, 2H); ^13^C NMR (150 MHz, CDCl_3_): *δ* 15.91 (15.94), 17.03 (17.05), 17.34 (17.36), 19.66 (19.68), 21.35 (21.37), 21.57 (21.61), 23.30 (23.33), 23.34 (23.37), 23.63 (23.65), 24.86 (24.99), 26.59 (26.69), 28.00 (28.09), 31.05 (31.11), 32.50, 34.12 (34.15), 34.24 (34.26), 36.38 (36.41), 37.63 (37.66), 38.10 (38.11), 39.09 (39.11), 39.15 (39.16), 39.80 (39.85), 42.63 (42.72), 46.37 (46.38), 46.86 (46.89), 47.98 (48.00), 53.86 (53.91), 55.29 (55.35), 106.24 (106.27), 110.17 (110.21), 117.76 (117.80), 122.36 (122.42), 126.08 (126.11), 128.01 (128.06), 128.50 (128.52), 134.60 (134.61), 139.41 (139.47), 141.15 (141.18), 178.37 (178.57); IR (KBr, cm^−1^): 3318, 2924, 2853, 1634, 1521, 1455, 1379, 1305, 1083, 963, 796; HRMS (ESI): *m*/*z* [M + H]^+^ calcd. for C_77_H_109_N_4_O_2_: 1121.8551; found: 1121.8558.

#### 4.4.3. *N*,*N*′-(butane-1,4-diyl)-bis(5′-methyl-1′*H*-ursa-2,12-dieno[3,2-*b*]indol-28-carboxamide) (**7c**)

Yellow solid; Yield: 63%; mp. 255–258 °C; ^1^H NMR (600 MHz, CDCl_3_): *δ* 0.86 (s, 3H), 0.87 (s, 3H), 0.89–0.92 (m, 12H), 0.94 (s, 3H), 0.96 (s, 3H), 1.07 (s, 3H), 1.08 (s, 3H), 1.13 (s, 3H), 1.15 (s, 3H), 1.24 (s, 3H), 1.25 (s, 3H), 1.30–2.08 (m, 38H), 2.08–2.25 (m, 6H), 2.31 (s, 3H), 2.32 (s, 3H), 2.68 (d, *J* = 14.7 Hz, 2H), 2.99 (m, 4H), 5.31 (brs, 2H), 6.78 (d, *J* = 8.2 Hz, 2H), 7.06 (s, 2H), 7.08–7.15 (m, 4H), 10.50 (d, *J* = 7.1 Hz, 2H); ^13^C NMR (150 MHz, CDCl_3_): *δ* 15.52, 16.72 (16.74), 17.12, 19.03 (19.10), 21.10, 21.28, 22.75 (22.76), 23.00 (23.01), 23.18 (23.19), 23.52 (23.64), 26.33 (26.41), 27.37 (27.41), 30.55 (30.56), 33.66, 33.80 (33.82), 36.12, 36.82, 37.05, 37.57, 38.45, 41.72 (41.81), 45.76, 46.04, 46.53, 46.65, 52.20, 53.01, 54.19, 104.23, 110.16, 117.08, 121.34, 124.76, 125.91, 127.77, 134.54, 138.45, 141.21, 176.04; IR (KBr, cm^−1^): 3313, 2925, 2853, 1635, 1523, 1455, 1379, 1306, 1087, 792, 732; HRMS (ESI): *m*/*z* [M + H]^+^ calcd. for C_78_H_111_N_4_O_2_: 1135.8707; found: 1135.8715.

### 4.5. Biological Evaluation

#### 4.5.1. In Vitro Anti-Proliferation Assay

Compounds **4a**–**f**, **5a**–**f**, **6a**–**f**, and **7a**–**c** were evaluated for their antitumor activity against two human hepatocarcinoma cells (HepG2 and SMMC-7721) and the human normal hepatocyte cell line (LO2) using the typical MTT assay according to the literature [31]. Cell lines were obtained from American Tissue Culture Collection (ATCC). Briefly, different tumor cells were grown in DMEM medium supplemented with 10% fetal bovine serum, penicillin (100 U/mL), and streptomycin (50 μg/mL). Cells were harvested at log phase of growth and seeded in 96-well plates (100 μL/well at a density of 2 × 10^5^ cells/mL). After 24 h incubation at 37 °C and 5% CO_2_ to allow cell attachment, cultures were exposed to various concentrations of the isolated compounds for 48 h. Finally, MTT solution (2.5 mg/mL in PBS) was added (40 μL/well). Plates were further incubated for 4 h at 37 °C, and the formazan crystals formed were dissolved by adding 150 μL/well of DMSO. Absorption at 570 nm was measured with an ELISA plate reader. The results were expressed as IC_50_ values, which was defined as the concentration at which 50% survival of cells was obtained. Doxorubicin, camptothecin, and etoposide (VP-16) were co-assayed as positive controls.

#### 4.5.2. Topoisomerase I Inhibition Assay

The activity of DNA topoisomerase I (TaKaRa, Kyoto, Japan) was determined by measuring the relaxation of supercoiled DNA pBR322 [32]. Camptothecin was used as a positive control. The reaction mixture was prepared according to the manufacture’s protocol, and incubated at 37 °C for 30 min. The reactions were terminated by adding dye solution containing 1% SDS, 0.02% bromophenol blue and 50% glycerol. The mixtures were applied to 1% agarose gel and subjected to electrophoresis for 1 h, in 1× TAE buffer (40 mM Tris-acetate, 1 mM EDTA). Gels were stained for 30 min in 60 mL 1× TAE buffer with ethidium bromide (0.5 μg/mL). DNA bands were visualized by transillumination with UV light and then photographed by a Gel Doc XR+ System (BIO-RAD, Hercules, CA, USA).

#### 4.5.3. Topoisomerase II Inhibition Assay

Human Topoisomerase IIα activity was detected by measuring the relaxation of supercoiled pBR322 plasmid DNA [22]. Reaction mixture contained 10 mM Tris (pH 6.9), 50 mM NaCl, 50 mM KCl, 5 mM MgCl_2_, 1 mM ATP, pBR322 DNA (100 ng), and 2 units of Topo IIα (USB, Affymetrix, Waltham, MA, USA) in a final volume of 20 μL. For inhibition studies, the compounds were pre-incubated with human Topo IIα and DNA for 15 min. Compounds were used at the appropriate concentrations by dissolving in 2% (*v*/*v*) DMSO. DMSO didn’t show detrimental effect on the enzyme activity at concentration up to 2% (*v*/*v*). Reaction mixture was incubated at 37 °C for 30 min and stopped by the addition of 7 mM EDTA. Reaction product was mixed with DNA loading dye and electrophoresed on 1% TAE-Agarose. The gel was stained with ethidium bromide (0.5 μg/mL) for 30 min, destained twice in 1× TAE buffer, and then visualized using a BIO-RAD Gel Doc XR+ System. Etoposide (VP-16) was co-assayed as a positive control.

#### 4.5.4. Molecular Docking

The molecular modeling of compound **5f** was performed with Schrödinger Suite 2015-1 (Schrödinger LLC., New York, NY, USA). The crystal structure of Topo IIα (PDB ID: 5GWK) was downloaded from Protein Data Bank (PDB) and prepared using the Protein Preparation Wizard workflow from Schrödinger Suite, including the optimization of hydrogen bond network and a short energy minimization with position restraints on heavy atoms using OPLS_2005 force field. The docking grid was generated according to the initial ligand etoposide. Then, the target compounds were freely docked into the designated binding site using the standard protocol implemented in Maestro v 10.1 (Schrödinger LLC, Cambridge, MA, USA). Van der Waals (vdW) scaling of 0.8 and partial cut-off of 0.15 were set to soften the potential for non-polar sites, and no constraints were specified. The best docked pose ranked by Glide Score value was recorded and saved for the ligand. The structures of complexes were analyzed for interaction modes, and the binding pose of compound **5f** with Topo IIα was displayed using the Discovery Studio 2016 client (Accelrys Software, San Diego, CA, USA).

#### 4.5.5. Cell Apoptosis Analysis

The extent of apoptosis was quantitatively measured using Annexin V-FITC/PI dual staining assay [33]. SMMC-7721 cells were seeded into a six-well plate at 5 × 10^5^ cells per well in 10% fetal calf serum (FBS)-DMEM into six-well plates and treated with different concentrations of the indicated compound **5f** for 48 h. The cells were detached with 0.25% trypsin, washed with ice-cold PBS for twice and then resuspended in 1 × Binding buffer (0.1 M Hepes/NaOH (pH 7.4), 1.4 M NaCl, 25 mM CaCl_2_). The cells were stained with 5 μL of Annexin V-FITC and 5 μL of PI (propidium indole) to each tube. The cells were gently vortexed and incubated in the dark at room temperature for 15 min and then kept at 4 °C. The samples were analyzed by a flow cytometer (Becton-Dickinson FACSCalibur, Franklin Lakes, NJ, USA), and data were analyzed using the FlowJo software (Becton-Dickinson Biosciences, Franklin Lakes, NJ, USA).

#### 4.5.6. Acridine Orange/Ethidium Bromide (AO/EB) Staining

SMMC-7721 cells were seeded into six-well plates and incubated at 37 °C with 5% CO_2_ for 24 h. Then, the cells were exposed to different concentrations of compound **5f** (0, 0.3, 0.6, and 1.2 μM) for 48 h. After that, 10 μL of each fluorescent dye cantaining AO and EB (10 μg/mL) were added to each well in equal volumes respectively and incubated for 5 min in the dark. The cells were visualized through an inverted fluorescence microscope (Olympus 1X71 Inverted System Microscope, Olympus, Tokyo, Japan) [26].

#### 4.5.7. ROS Generation Assay

ROS generation assay was performed by using the reactive oxygen species assay kit (Beyotime Biotech., Shanghai, China). Intracellular ROS generation was tested through dichlorodihydro fluorescein diacetate (DCFH-DA) assay [34]. DCFH-DA is taken up by SMMC-7721 cells, and then activated by esterase-mediated cleavage of acetate to form dichlorodihydro fluorescein (DCFH), which is trapped in the cells. DCFH is converted to fluorescein DCF in the presence of ROS. SMMC-7721 cells were seeded in six-well plates and incubated with different concentrations of compound **5f** for 48 h. After removing the compound solution, cells were treated with 10 μM of DCFH-DA at 37 °C for 20 min. Subsequently, the cells were washed with PBS for three times and then exposed to light. Immediately after light exposure, the fluorescence intensity of dichlorofluorescein (DCF) was measured with excitation at 488 nm and emission at 525 nm by a flow cytometry (Becton-Dickinson FACSCalibur, Franklin Lakes, NJ, USA).

#### 4.5.8. JC-1 Mitochondrial Membrane Potential Assay

The JC-1 mitochondrial membrane potential assay kit (Keygene Biotech., Nanjing, China) was employed to measure mitochondrial depolarization in SMMC-7721 cells. The fluorescence probe JC-1 was used to detect the change of mitochondrial membrane potential [35]. Briefly, cells cultured in six-well plates after indicated treatments by compound **5f** were incubated with an equal volume of JC-1 staining solution (5 μg/mL) at 37 °C for 20 min and rinsed twice with PBS. Mitochondrial membrane potentials were monitored by determining the relative amounts of dual emissions from mitochondrial JC-1 monomers or aggregates using flow cytometry (Becton-Dickinson FACSCalibur, Franklin Lakes, NJ, USA). Mitochondrial membrane depolarization is indicated by an increase in the percentage of cells with low ΔΨm (green fluorescence, lower right quadrant) compared with cells with high ΔΨm (red fluorescence, upper right quadrant).

## Figures and Tables

**Table 1:** The cytotoxicity Topo I and II inhibitory activities of the target compounds.

	IC_50_ Values (μM)			Topo I Inhibition (%)		Topo II Inhibition (%)	
	SMMC-7721	HepG2	LO2	100 μM	20 μM	100 μM	20 μM
**4a**	3.50 ± 0.61	3.31 ± 0.35	29.59 ± 2.12	15.1	5.6	69.7	25.9
**4b**	4.42 ± 0.71	4.11 ± 0.55	31.06 ± 2.63	3.7	-^1^	68.0	36.9
**4c**	2.10 ± 0.21	2.24 ± 0.32	26.39 ± 1.32	0	-	68.7	38.3
**4d**	1.75 ± 0.11	1.47 ± 0.12	16.03 ± 1.07	13.0	0	71.7	32.7
**4e**	7.82 ± 0.78	10.72 ± 2.09	>50	0	-	63.3	26.8
**4f**	1.24 ± 0.16	1.35 ± 0.17	18.27 ± 0.78	0	-	73.7	31.3
**5a**	2.02 ± 0.21	2.45 ± 0.38	21.32 ± 1.59	24.5	12.4	72.3	31.2
**5b**	1.08 ± 0.22	1.26 ± 0.17	20.65 ± 1.73	34.2	9.0	72.5	38.2
**5c**	1.15 ± 0.14	1.20 ± 0.24	15.85 ± 1.06	0	-	75.8	38.9
**5d**	0.89 ± 0.11	1.04 ± 0.09	12.82 ± 0.83	0	-	81.1	41.3
**5e**	3.75 ± 0.37	5.27 ± 0.67	28.62 ± 0.76	0	-	70.8	31.5
**5f**	0.56 ± 0.08	0.91 ± 0.13	10.58 ± 0.52	0	-	83.5	43.2
**6a**	1.21 ± 0.19	1.32 ± 0.21	15.23 ± 1.15	28.8	13.7	71.1	27.0
**6b**	2.43 ± 0.21	2.91 ± 0.18	18.19 ± 1.64	15.1	0	69.0	31.7
**6c**	3.62 ± 0.23	3.45 ± 0.25	28.42 ± 2.98	16.8	0	70.0	35.2
**6d**	2.72 ± 0.31	2.31 ± 0.21	18.65 ± 1.36	0	-	72.5	32.8
**6e**	4.55 ± 1.56	7.84 ± 2.31	35.39 ± 3.13	0	-	65.6	28.2
**6f**	0.65 ± 0.07	1.01 ± 0.12	13.72 ± 1.28	0	-	81.8	39.8
**7a**	>50	>50	>50	0	-	23.8	9.7
**7b**	>50	>50	>50	2.0	-	17.8	9.8
**7c**	>50	>50	>50	0	-	16.9	8.1
CPT ^2^	0.56 ± 0.18	1.12 ± 0.26	8.93 ± 0.52	75.1	38.2	-	-
VP-16	0.36 ± 0.10	0.57 ± 0.16	5.92 ± 0.29	-	-	88.5	47.8
DOX	0.68 ± 0.11	0.81 ± 0.19	5.32 ± 0.67	-	-	-	-

^1^ -: Not tested. ^2^ CPT: camptothecin; VP-16: etoposide; DOX: doxorubicin.

## Supplementary Materials

Supplementary materials can be found at https://www.mdpi.com/1422-0067/21/8/2876/s1.

## Abbreviations

Topo	Topoisomerase
UA	Ursolic acid
HOBt	1-Hydroxybenzotriazole
DCC	*N*,*N*′-Dicyclohexylcarbodiimide
MTT	3-(4,5-Dimethyl-2-thiazolyl)-2,5-diphenyl-2-*H*-tetrazolium bromide
PI	Propidium Iodide
AO	Acridine orange
EB	Ethidium bromide
ROS	Reactive oxygen species
EDTA	Ethylene diamine tetraacetic acid
PBS	Phosphate buffer saline
SDS	Sodium dodecyl sulfate
