# Supplementary material for: Design, Synthesis, and Anticancer Evaluation of Novel Indole Derivatives of Ursolic Acid as Potential Topoisomerase II Inhibitors
Source: Int J Mol Sci. 2020 Apr 20;21(8):2876. doi: 10.3390/ijms21082876 (PMC7215373; doi:10.3390/ijms21082876)
Supplement: Supplementary file 1 [file ijms-21-02876-s001.pdf]

Supplementary Material

Design, Synthesis and Anticancer Evaluation of Novel Indole Derivatives of Ursolic Acid as Potential Topoisomerase II Inhibitors

A-Liang Li, Yun Hao, Wen-Yan Wang, Qing-Song Liu, Yue Sun and Wen Gu *

Jiangsu Provincial Key Lab for the Chemistry and Utilization of Agro-forest Biomass, Jiangsu Key Lab of Biomass-based Green Fuels and Chemicals, Co-Inovation Center for Efficient Processing and Utilization of Forest Products, College of Chemical Engineering, Nanjing Forestry University, Nanjing 210037, P. R. China;

* Correspondence: njguwen@163.com; (W.G.)

Figure S1~Figure S42. ^{1}H and ^{13}C NMR spectra of compounds 4a-f, 5a-f, 6a-f and 7a-c.

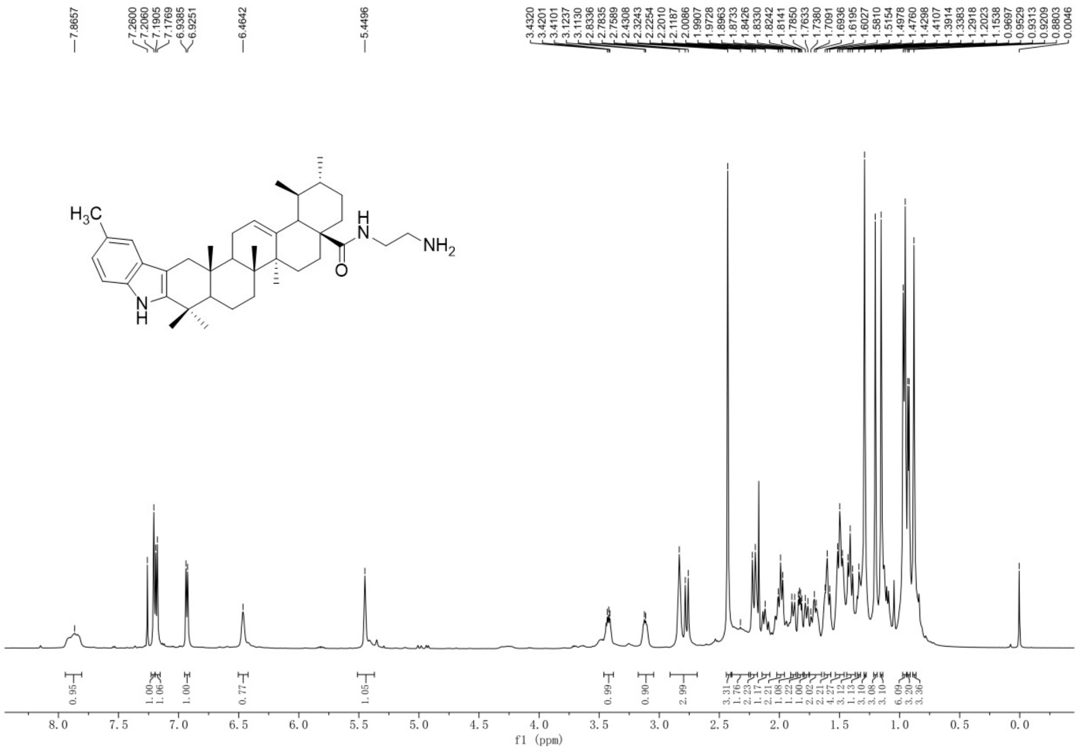

Figure S1. ^{1}H-NMR spectrum of compound **4a** (600 MHz, $CDCl_3$).

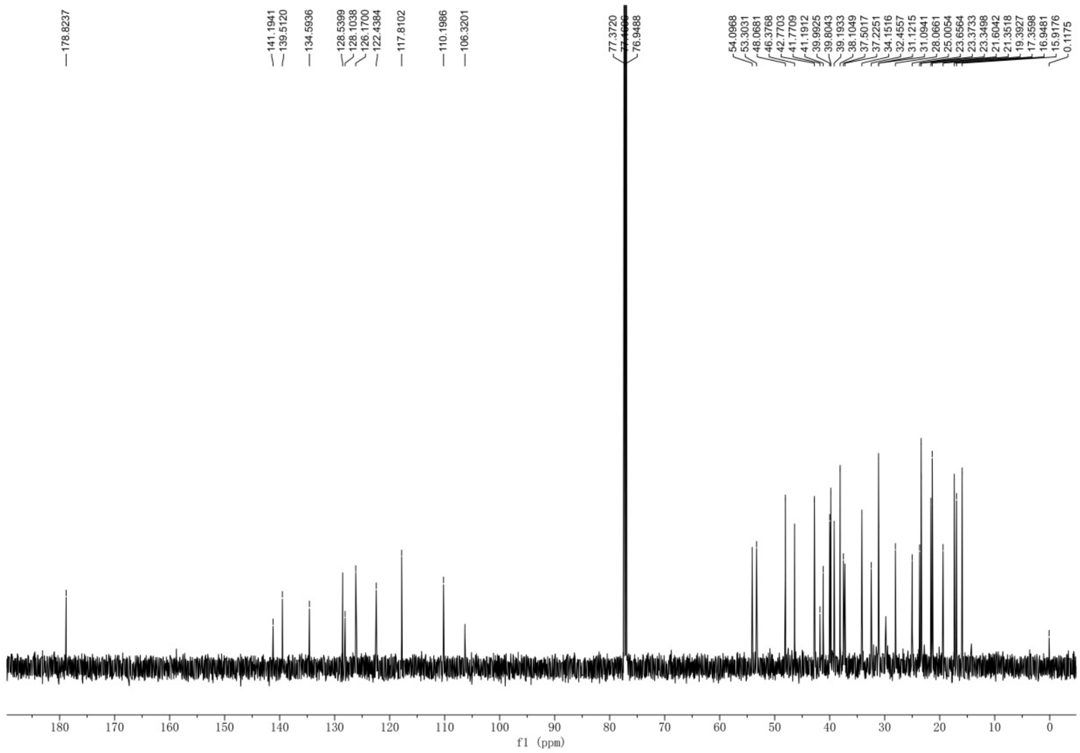

Figure S2. ^{13}C-NMR spectrum of compound **4a** (150 MHz, $CDCl_3$).

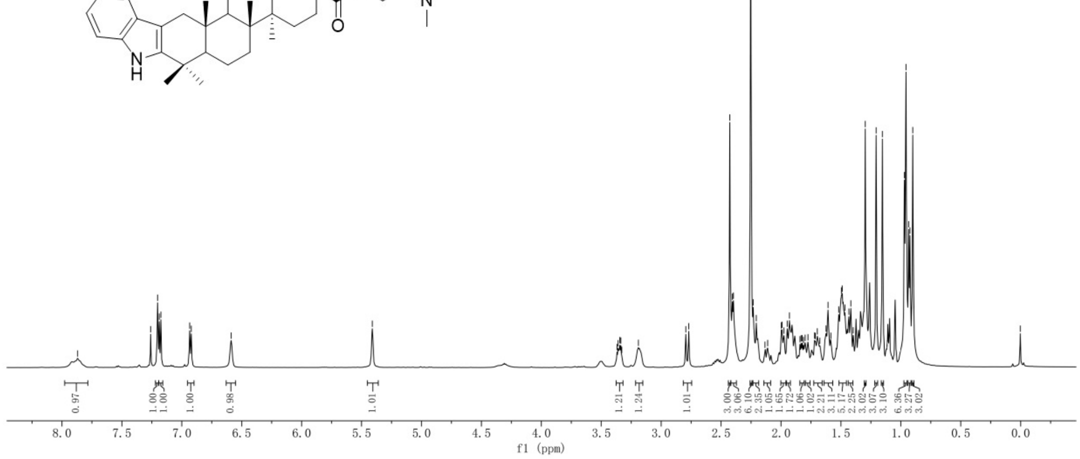

Figure S3. ^{1}H-NMR spectrum of compound **4b** (600 MHz, $CDCl_3$).

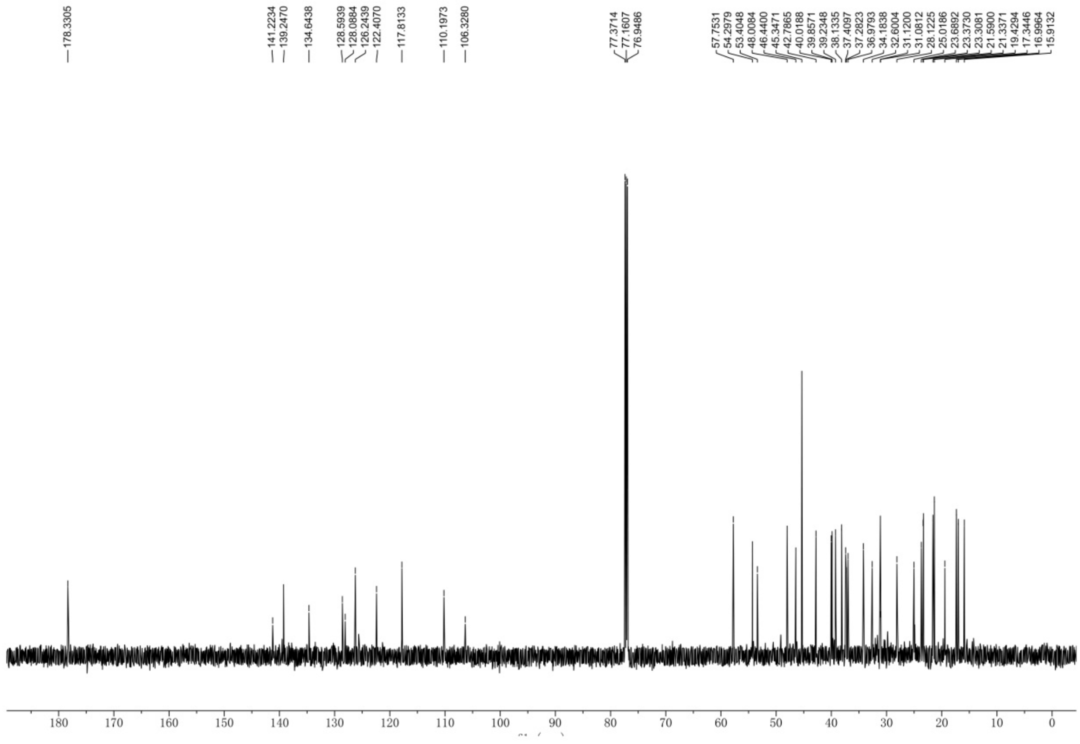

Figure S4. ^{13}C-NMR spectrum of compound **4b** (150 MHz, $CDCl_3$).

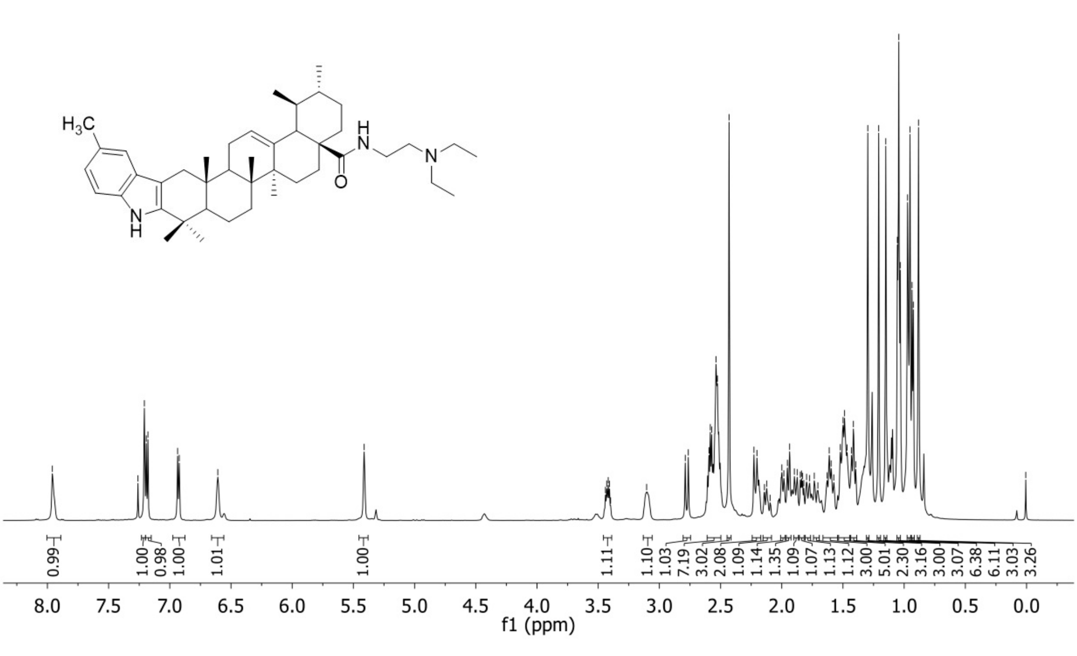

Figure S5. ^{1}H-NMR spectrum of compound **4c** (600 MHz, $CDCl_3$).

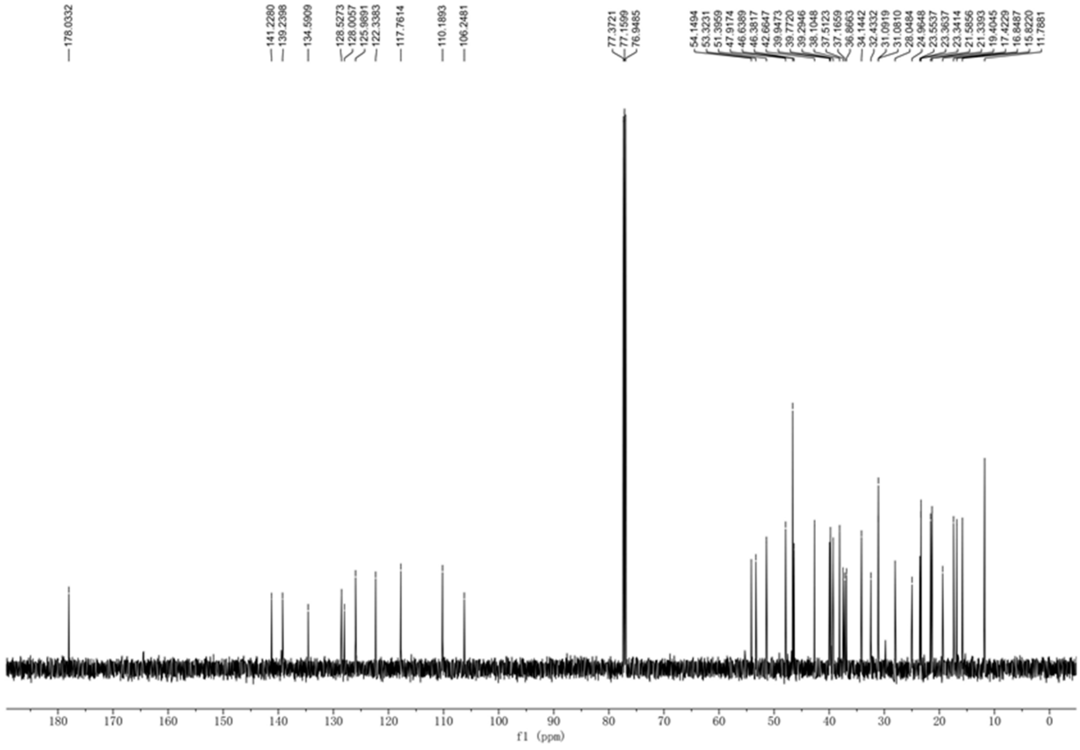

Figure S6. ^{13}C-NMR spectrum of compound **4c** (150 MHz, $CDCl_3$).

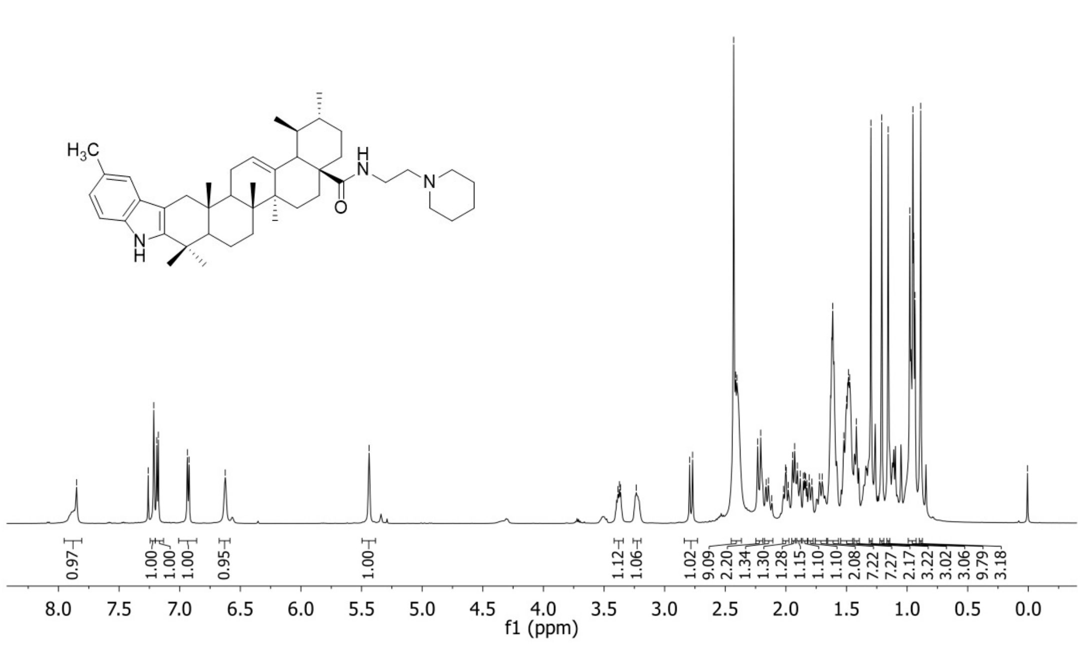

Figure S7. ^{1}H-NMR spectrum of compound **4d** (600 MHz, $CDCl_3$).

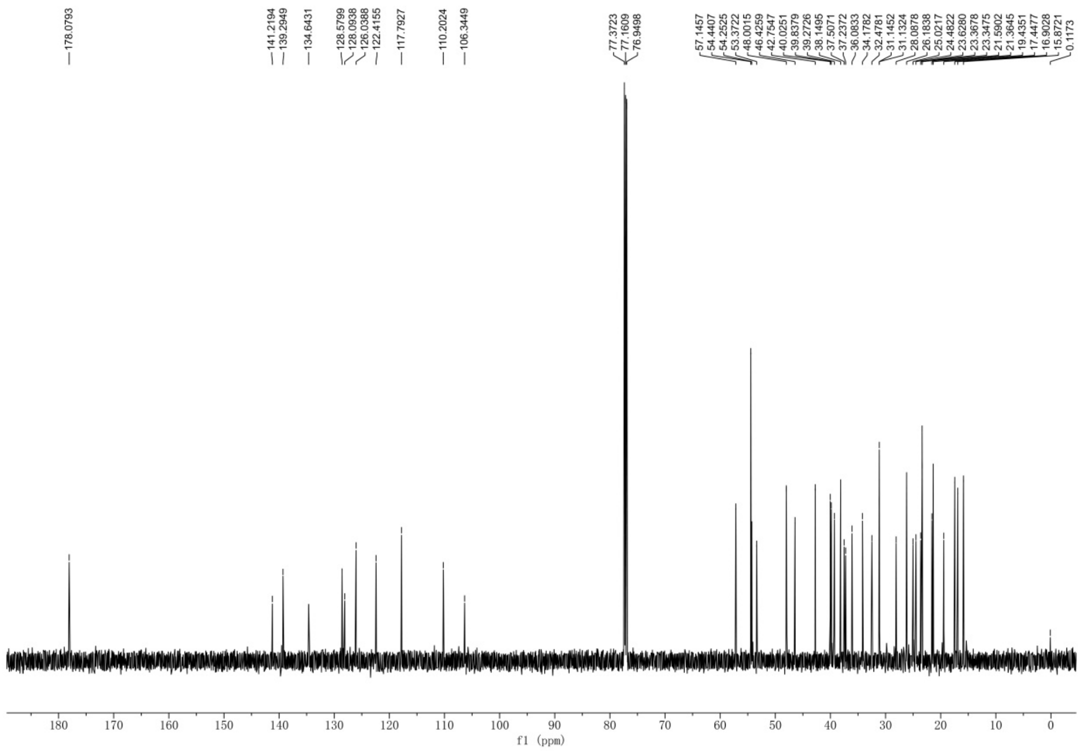

Figure S8. ^{13}C-NMR spectrum of compound **4d** (150 MHz, $CDCl_3$).

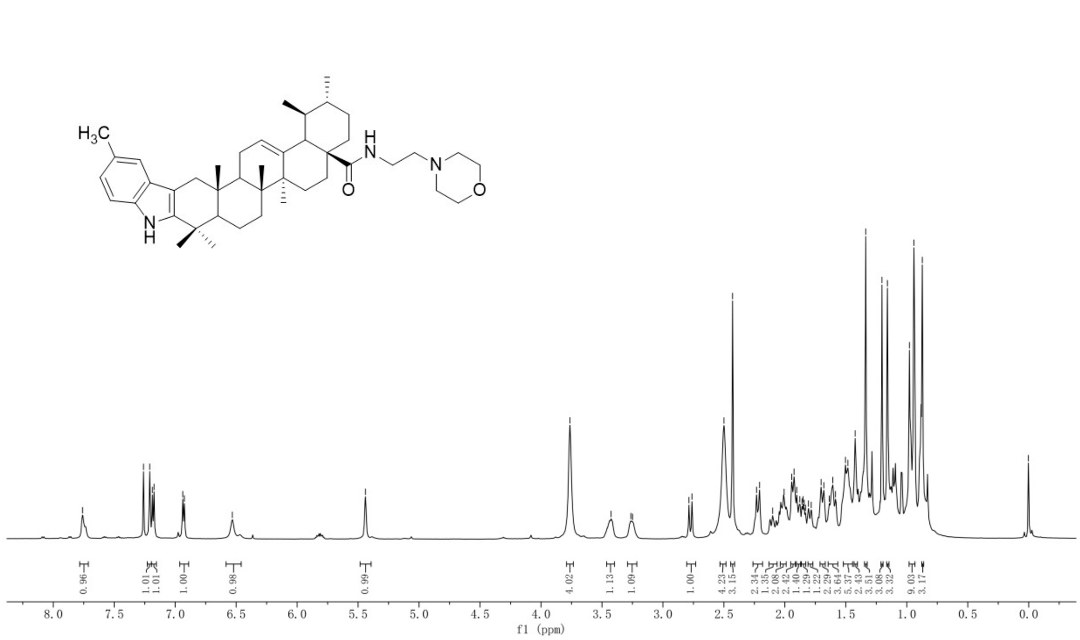

Figure S9. ^{1}H-NMR spectrum of compound **4e** (600 MHz, $CDCl_3$).

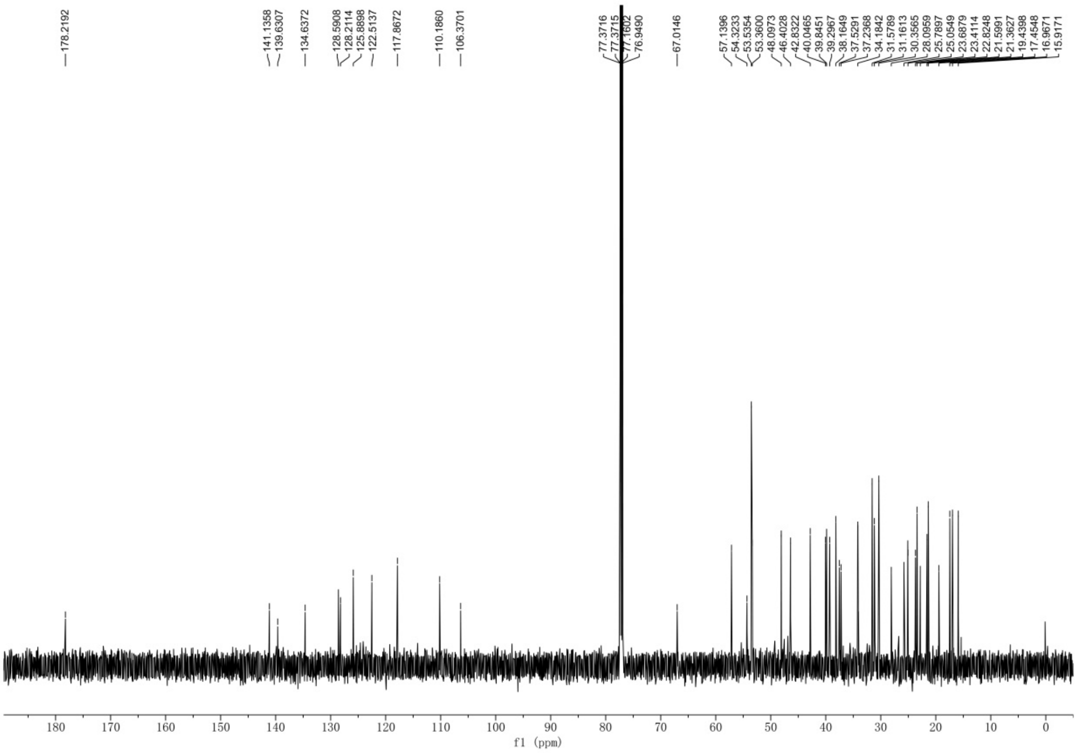

Figure S10. ^{13}C-NMR spectrum of compound **4e** (150 MHz, $CDCl_3$).

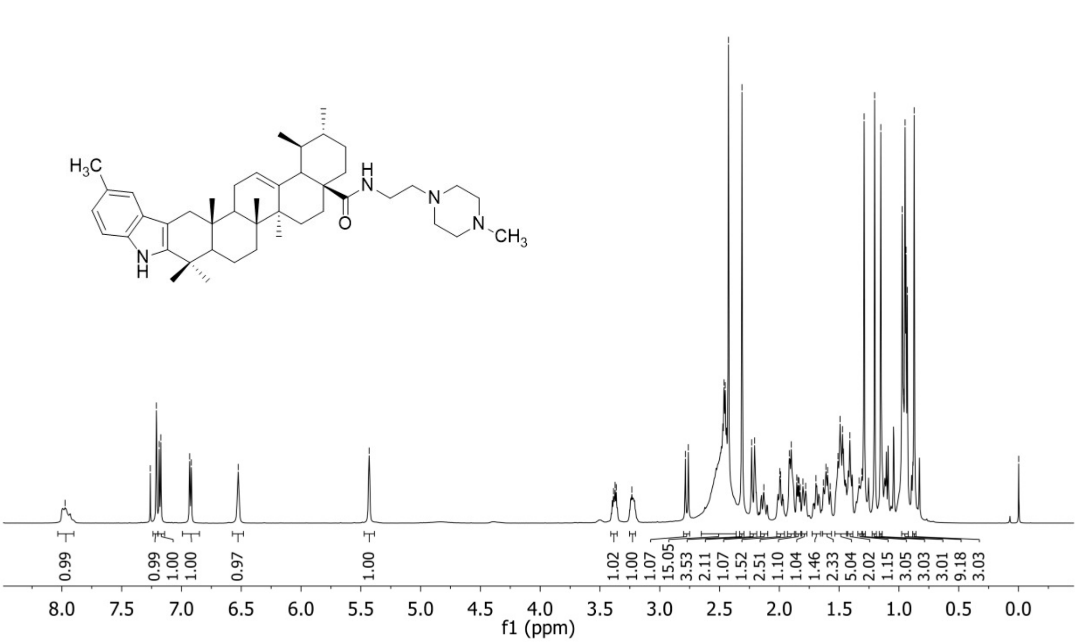

Figure S11. ^{1}H-NMR spectrum of compound **4f** (600 MHz, $CDCl_3$).

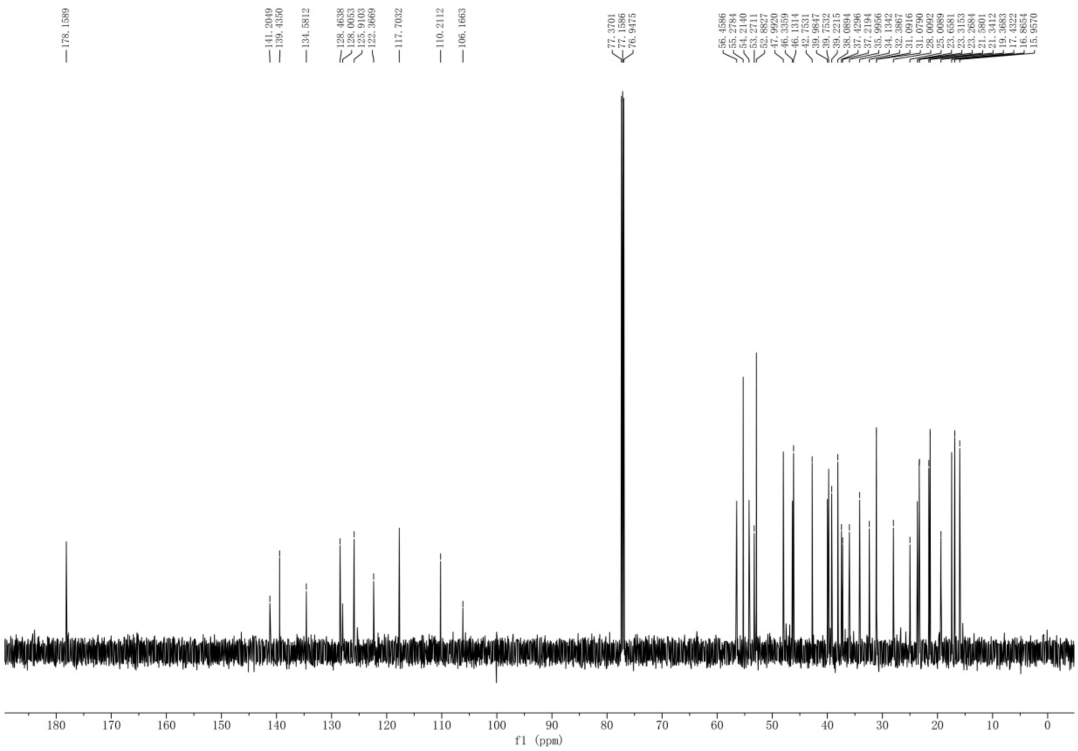

Figure S12. ^{13}C-NMR spectrum of compound **4f** (150 MHz, $CDCl_3$).

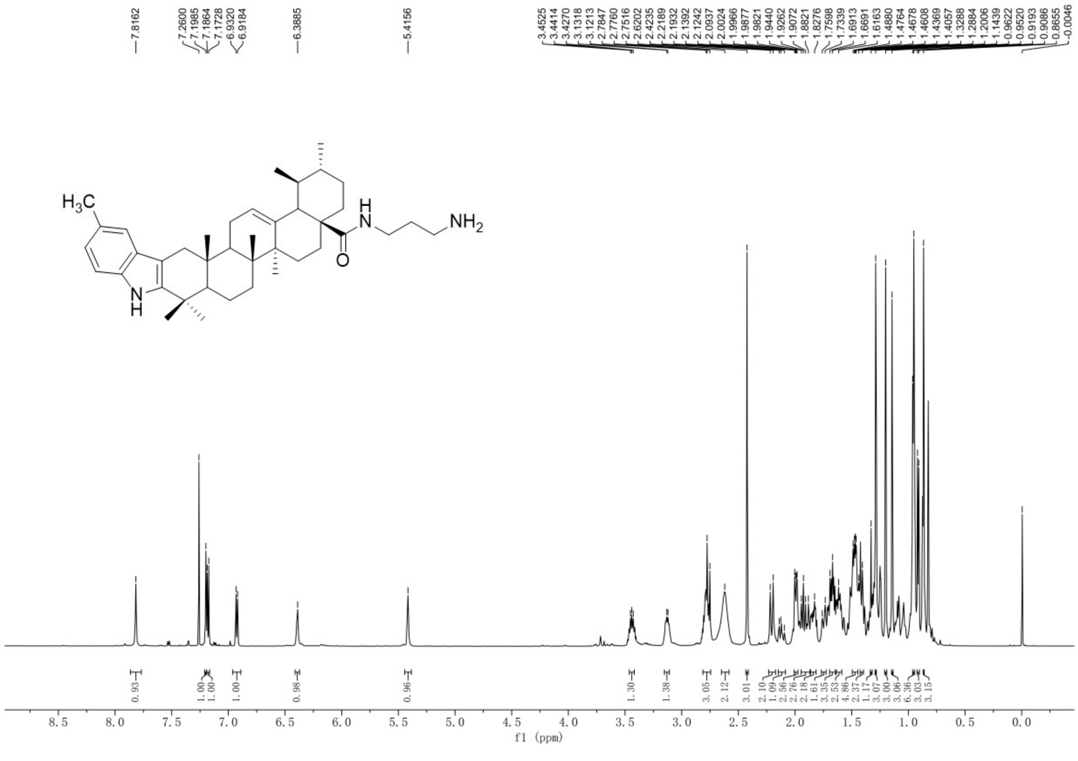

Figure S13. ^{1}H-NMR spectrum of compound **5a** (600 MHz, $CDCl_3$).

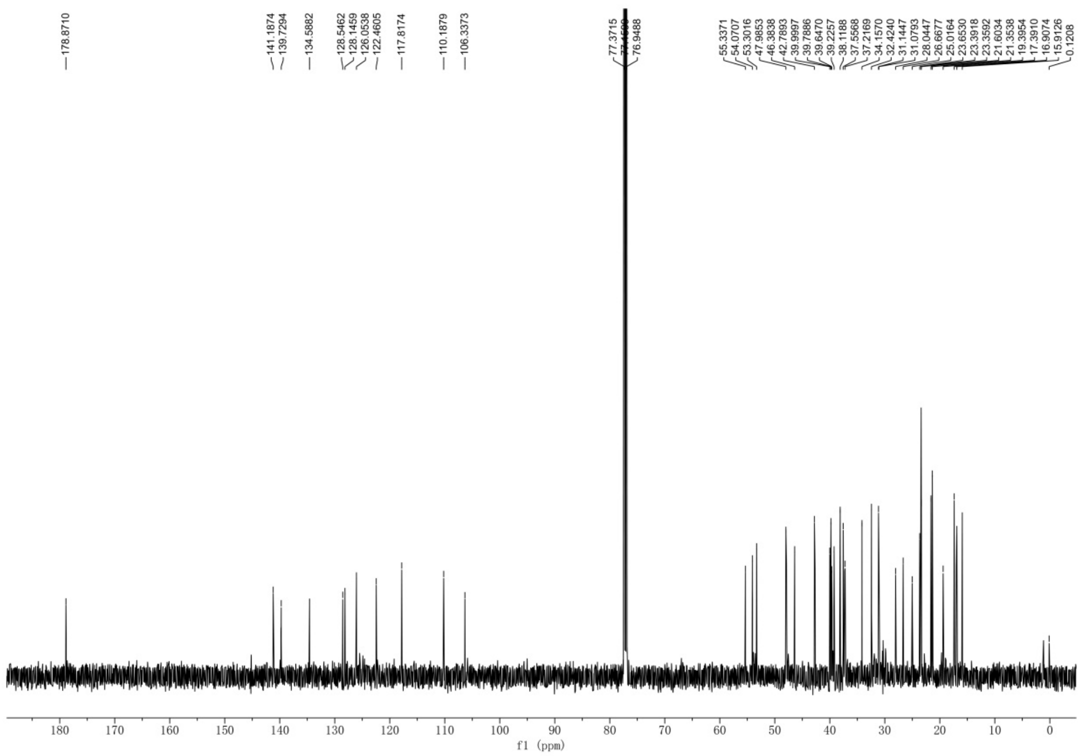

Figure S14. ^{13}C-NMR spectrum of compound **5a** (150 MHz, $CDCl_3$).

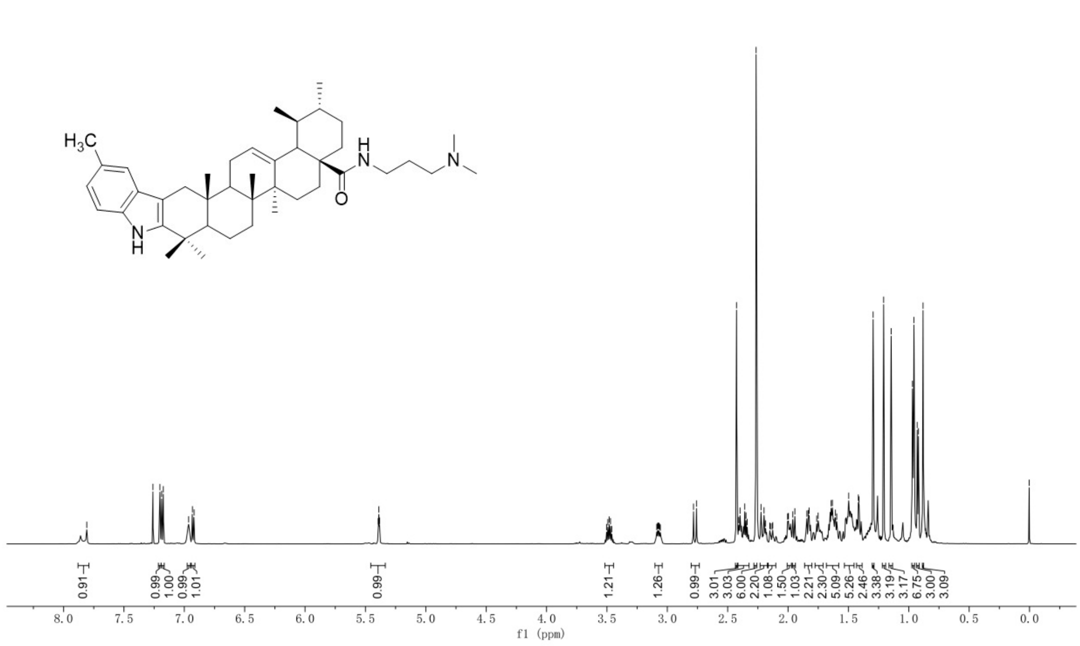

Figure S15. ^{1}H-NMR spectrum of compound **5b** (600 MHz, $CDCl_3$).

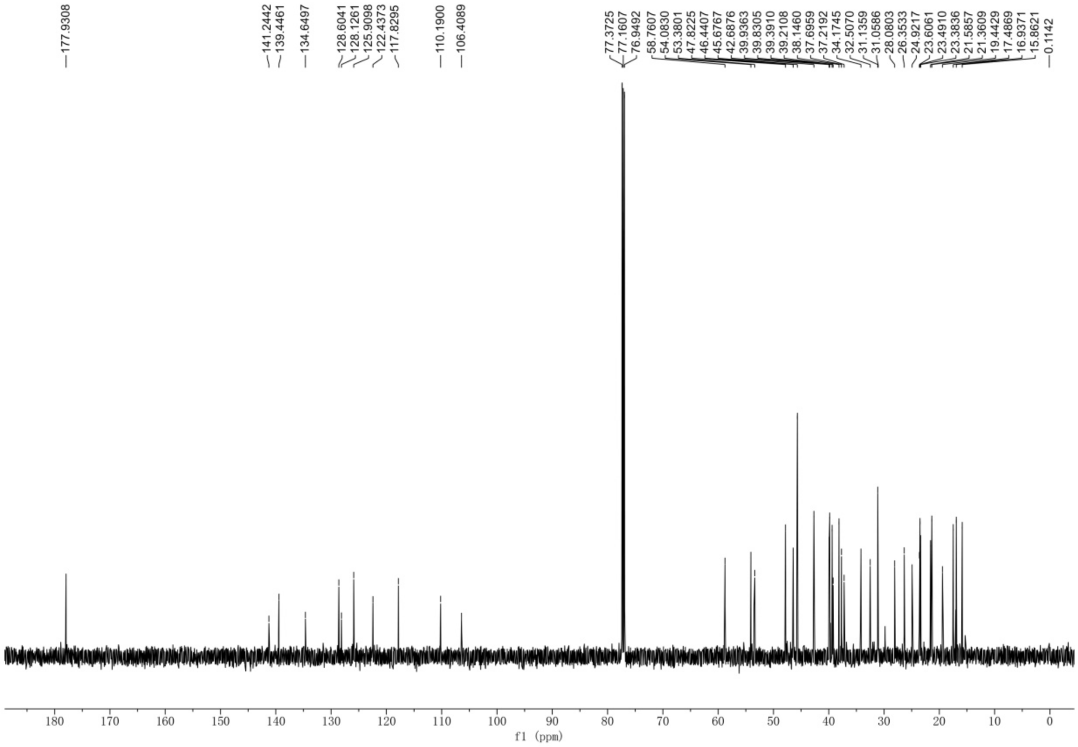

Figure S16. ^{13}C-NMR spectrum of compound **5b** (150 MHz, $CDCl_3$).

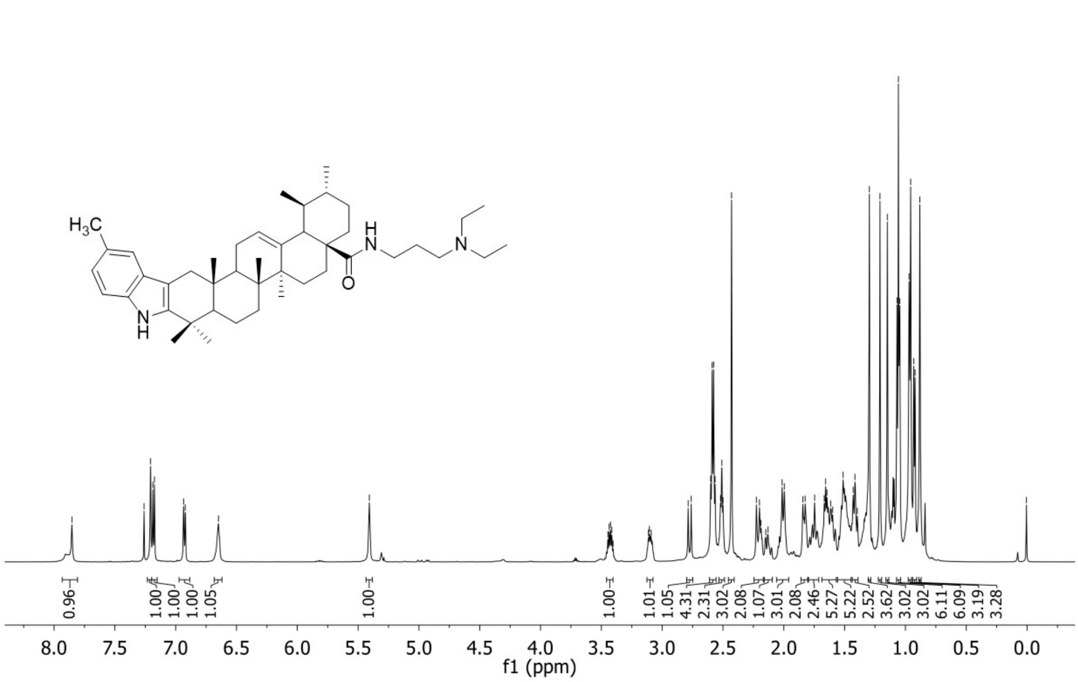

Figure S17. ^{1}H-NMR spectrum of compound **5c** (600 MHz, $CDCl_3$).

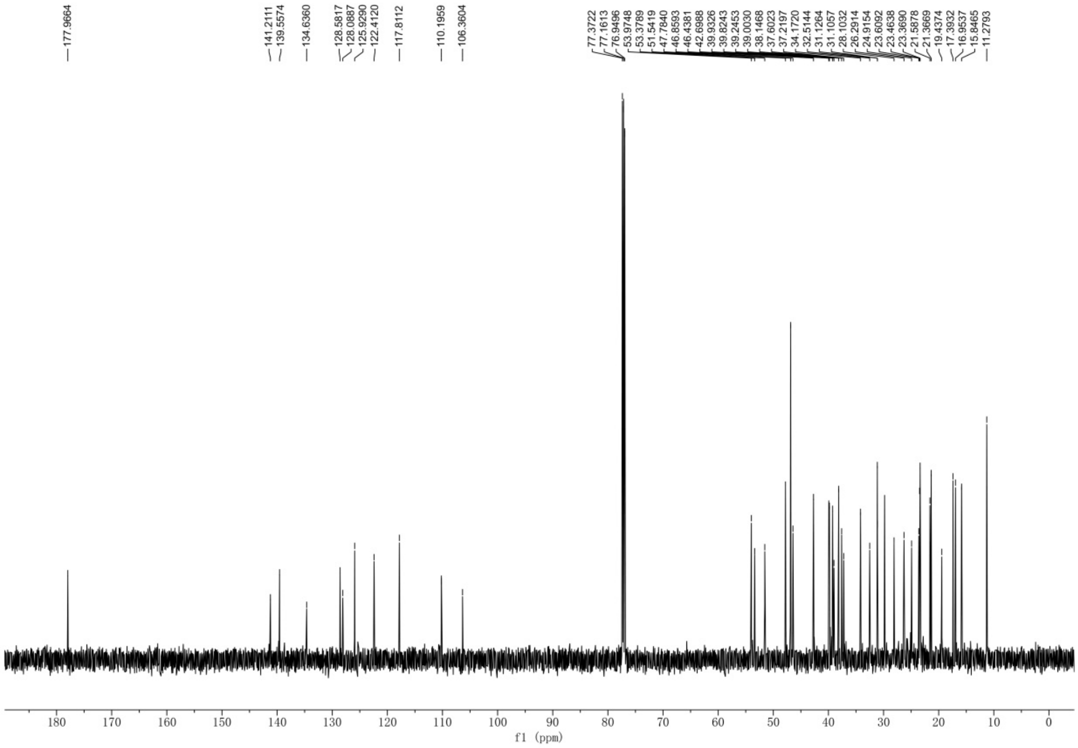

Figure S18. ^{13}C-NMR spectrum of compound **5c** (150 MHz, $CDCl_3$).

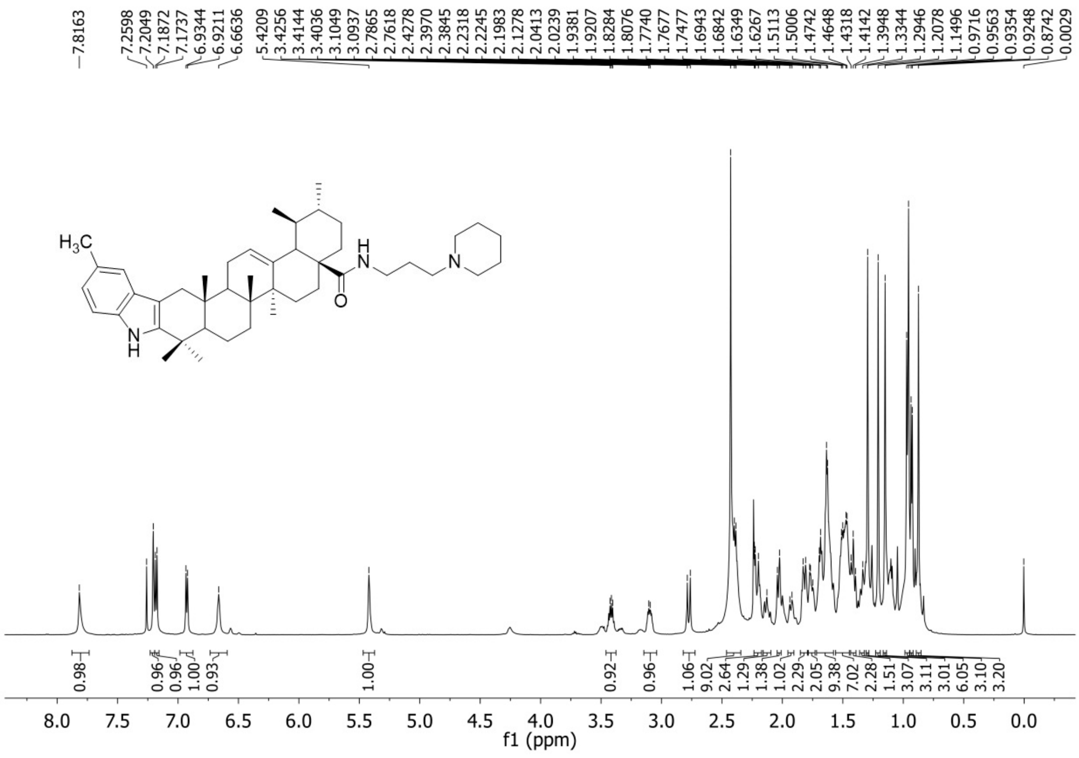

Figure S19. ^{1}H-NMR spectrum of compound **5d** (600 MHz, $CDCl_3$).

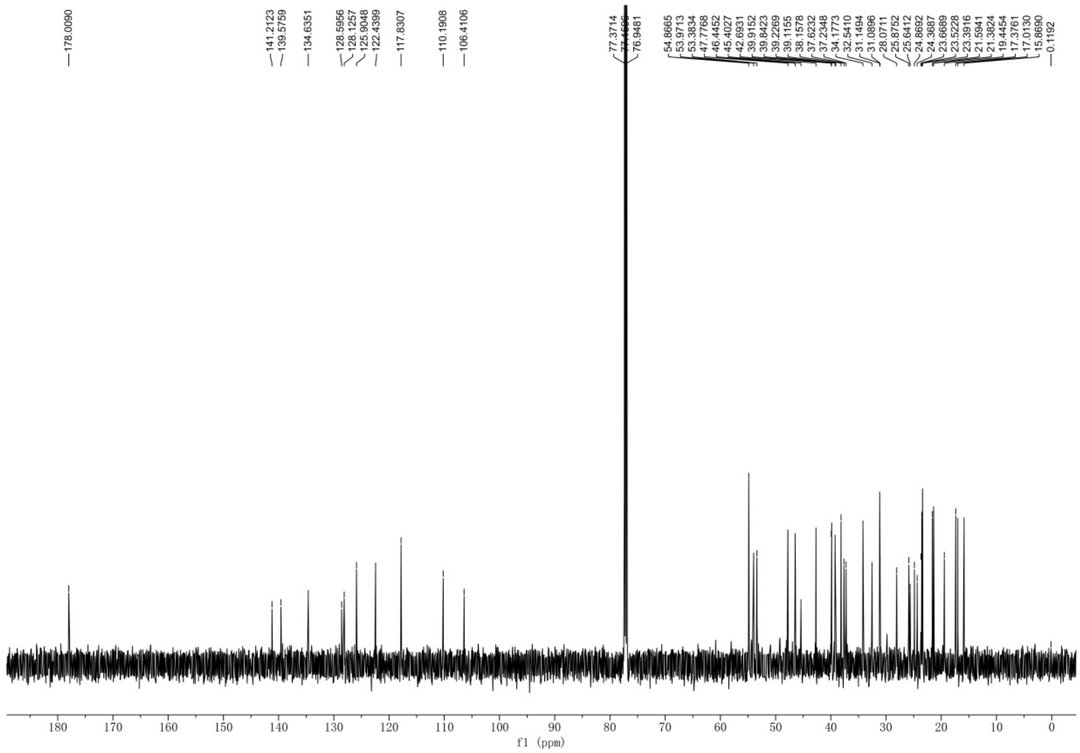

Figure S20. ^{13}C-NMR spectrum of compound **5d** (150 MHz, $CDCl_3$).

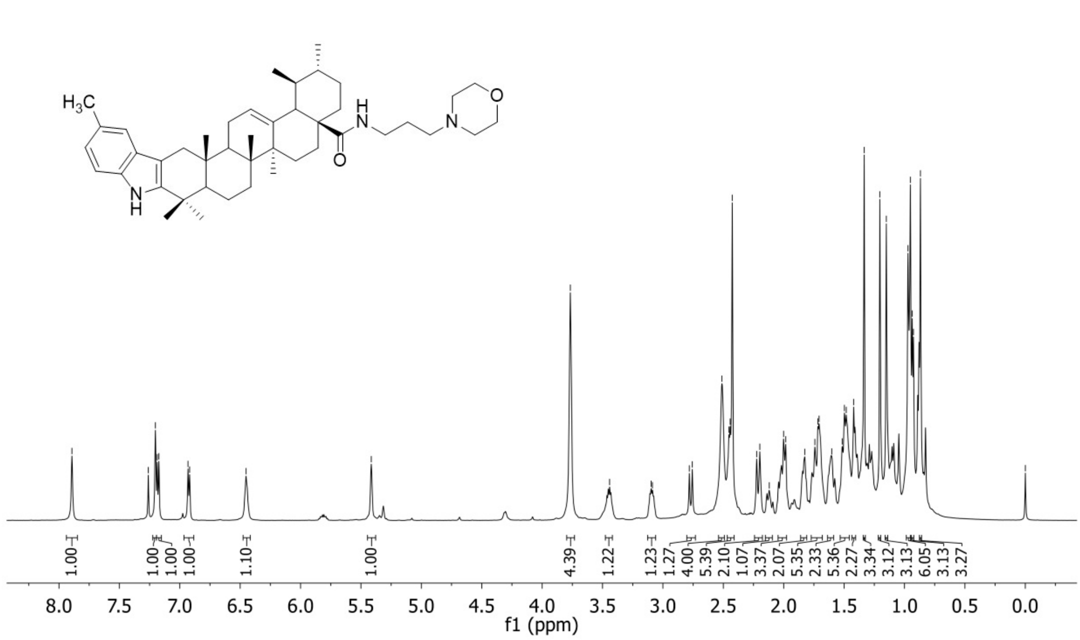

Figure S21. ^{1}H-NMR spectrum of compound **5e** (600 MHz, $CDCl_3$).

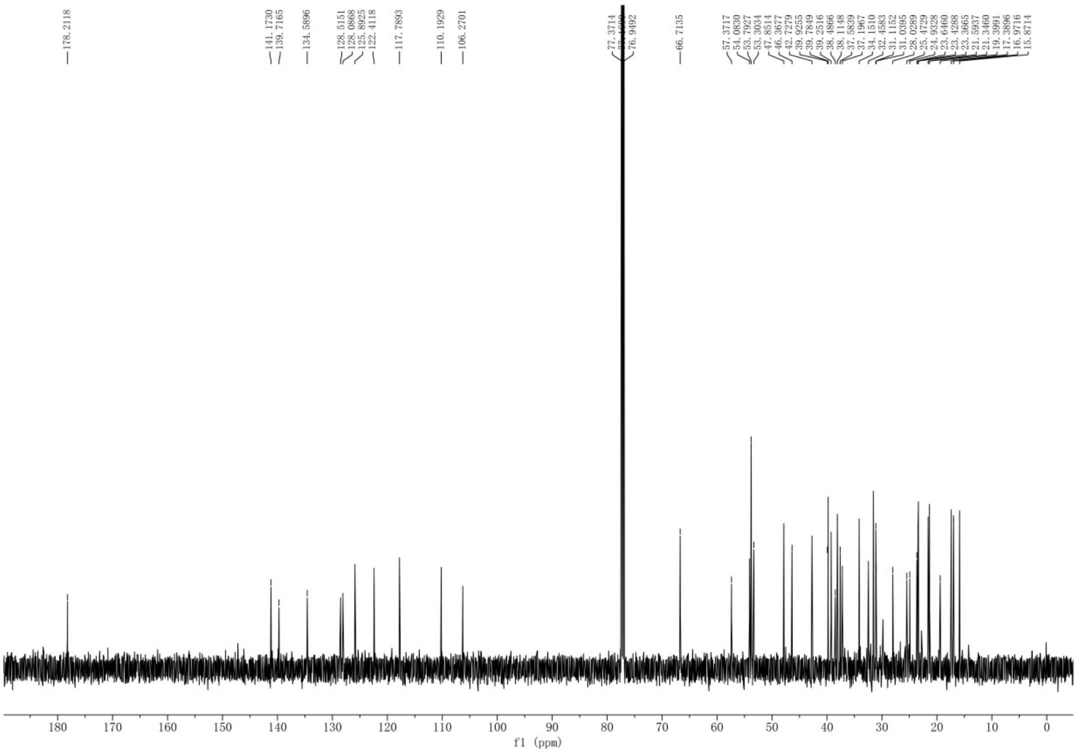

Figure S22. ^{13}C-NMR spectrum of compound **5e** (150 MHz, $CDCl_3$).

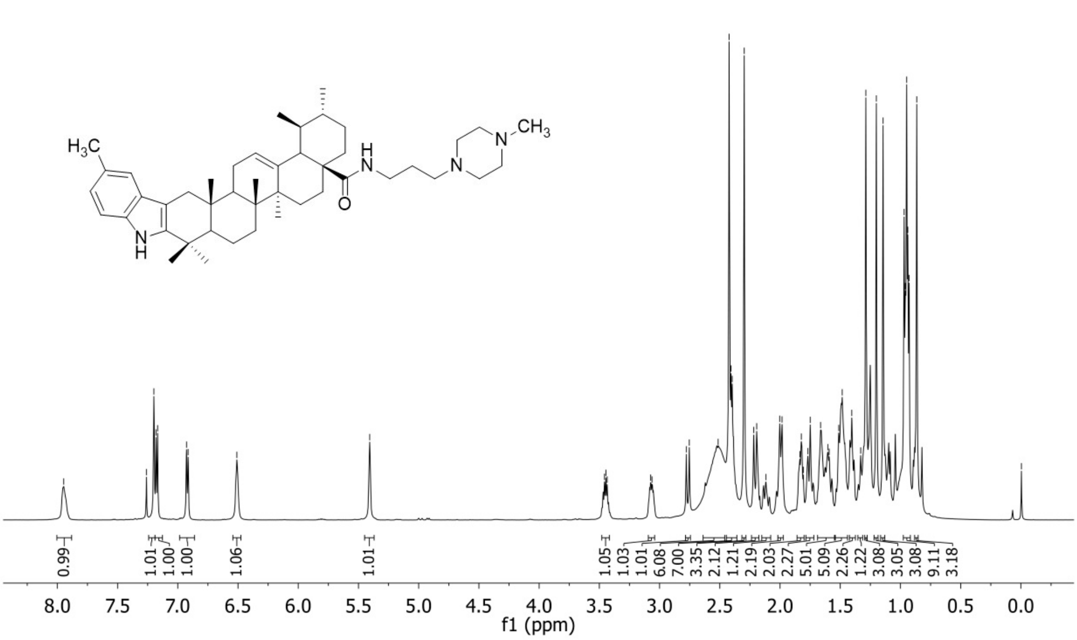

Figure S23. ^{1}H-NMR spectrum of compound **5f** (600 MHz, $CDCl_3$).

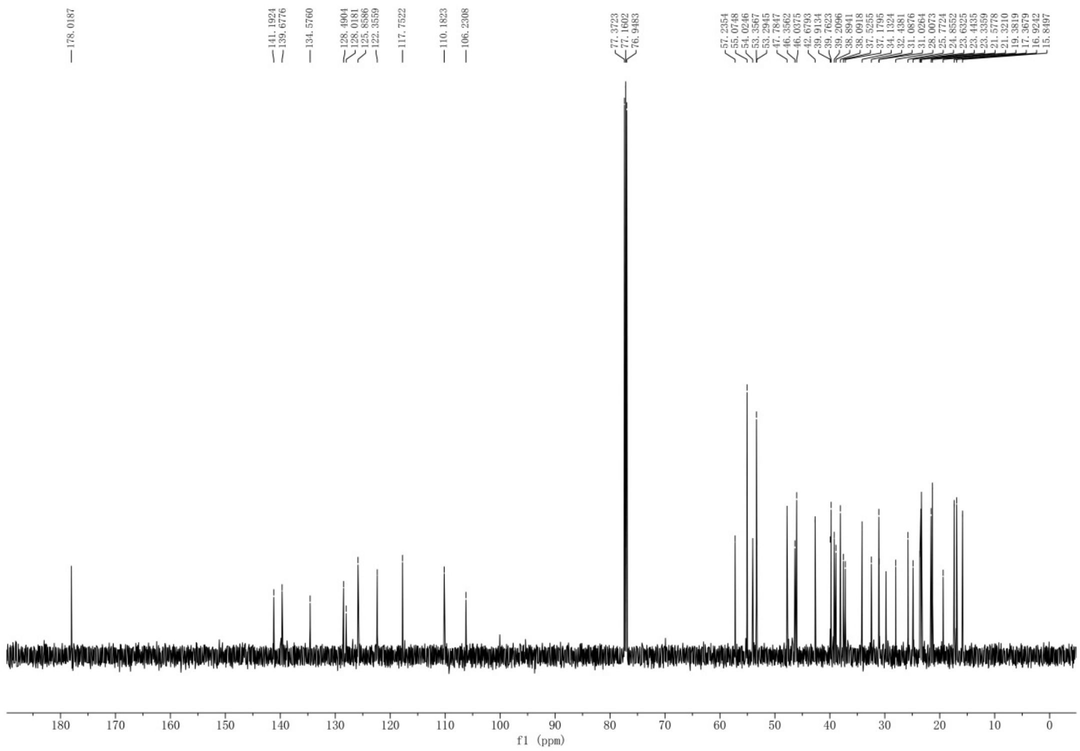

Figure S24. ^{13}C-NMR spectrum of compound **5f** (150 MHz, $CDCl_3$).

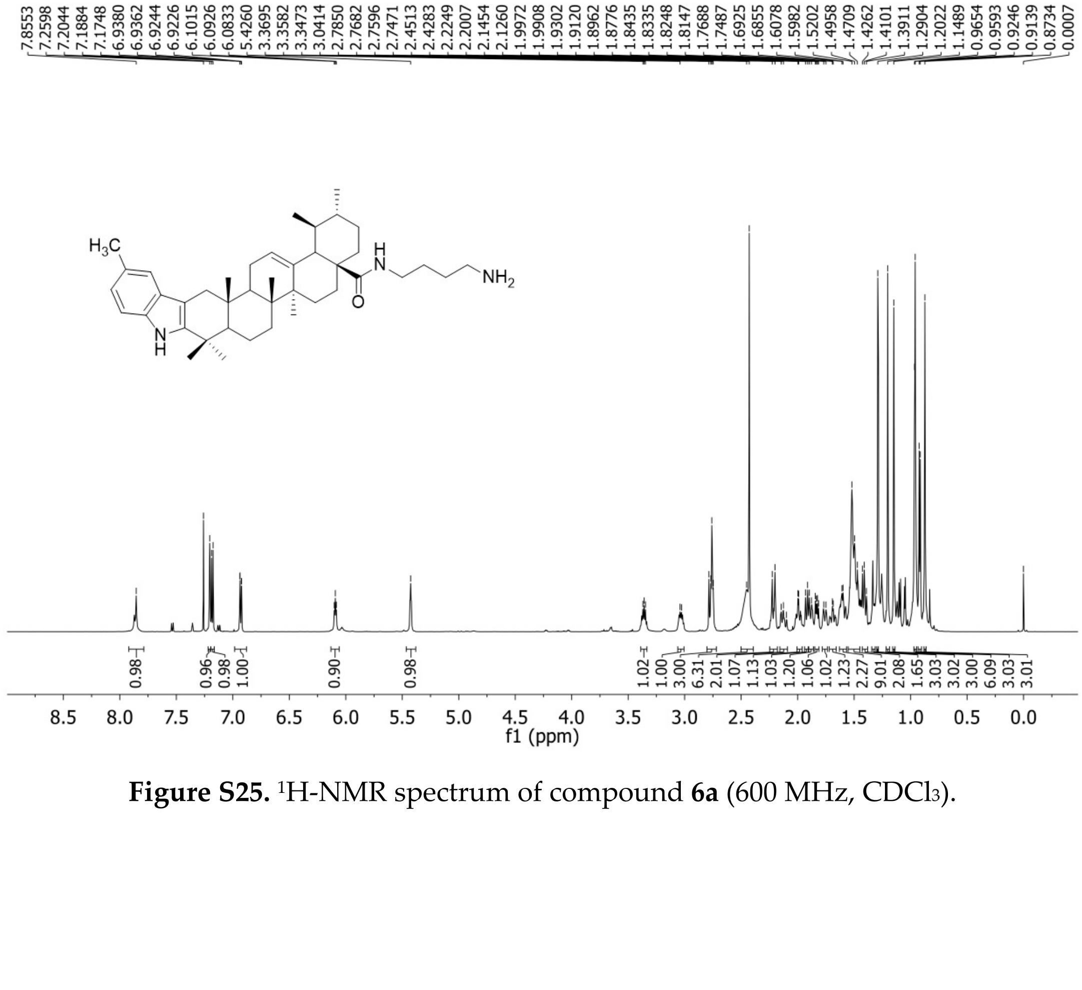

Figure S25. ^{1}H-NMR spectrum of compound **6a** (600 MHz, $CDCl_3$).

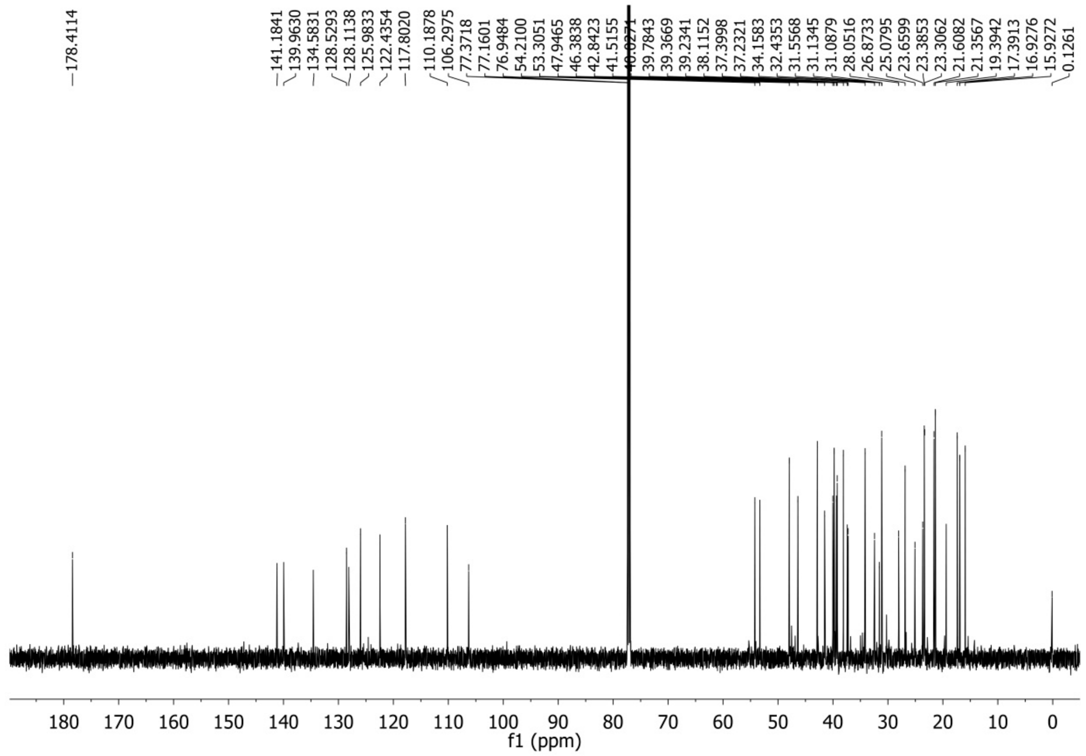

Figure S26. ^{13}C-NMR spectrum of compound **6a** (150 MHz, $CDCl_3$).

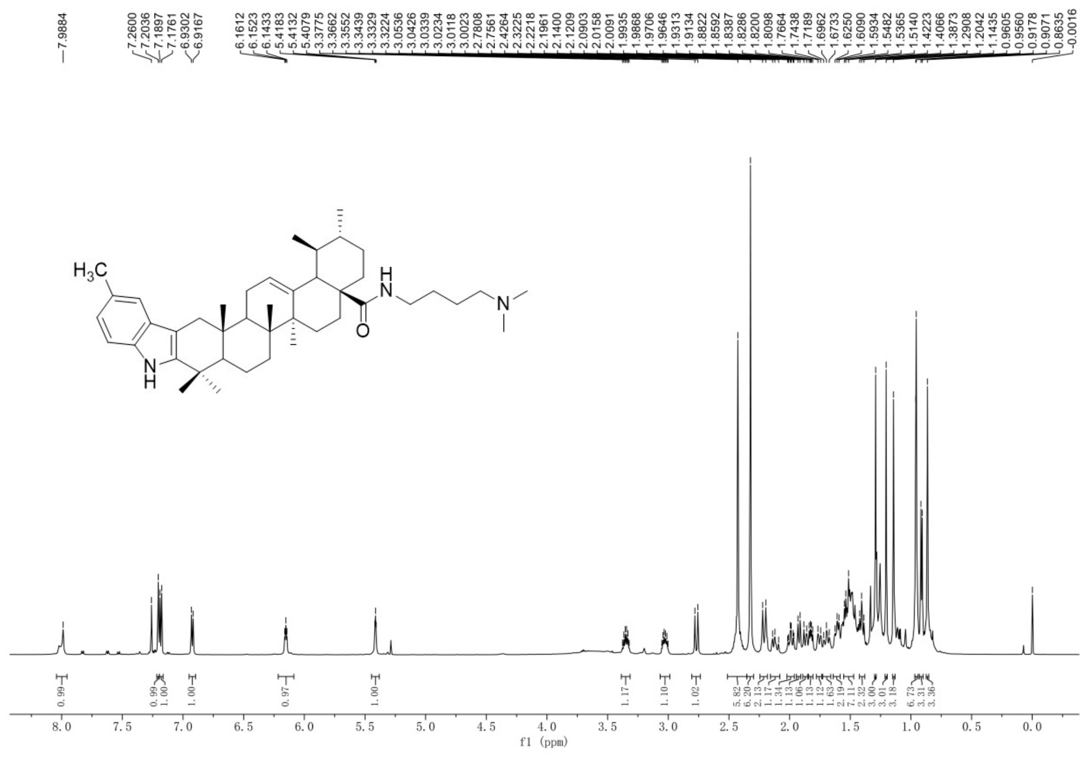

Figure S27. ^{1}H-NMR spectrum of compound **6b** (600 MHz, $CDCl_3$).

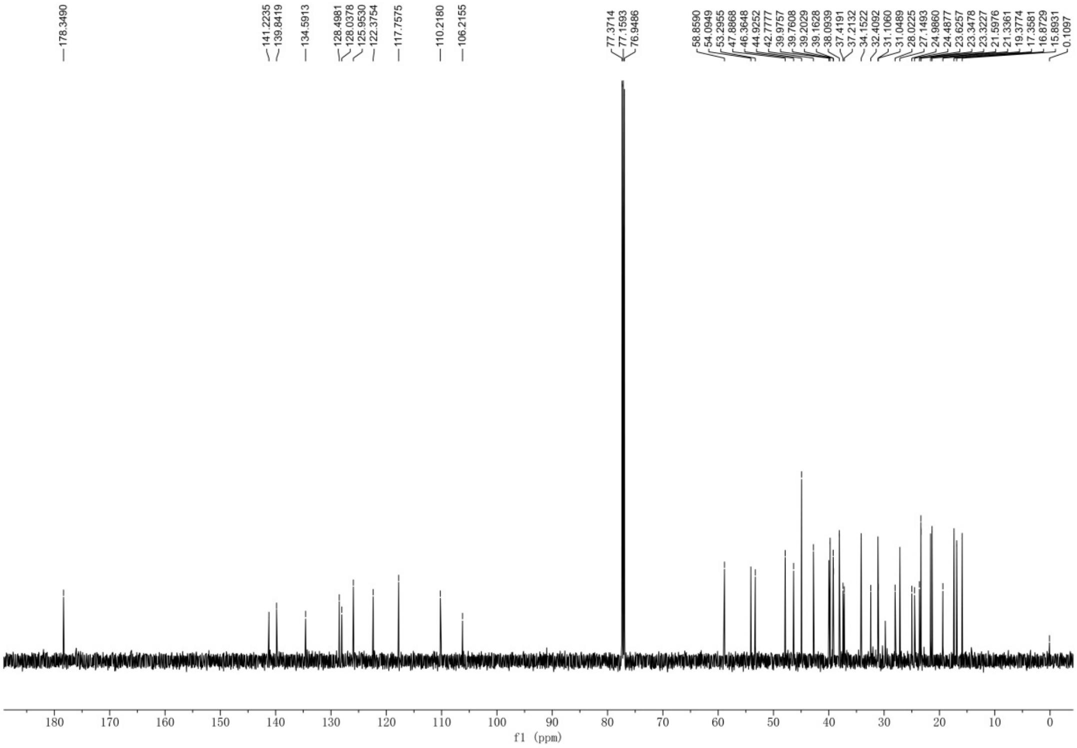

Figure S28. ^{13}C-NMR spectrum of compound **6b** (150 MHz, $CDCl_3$).

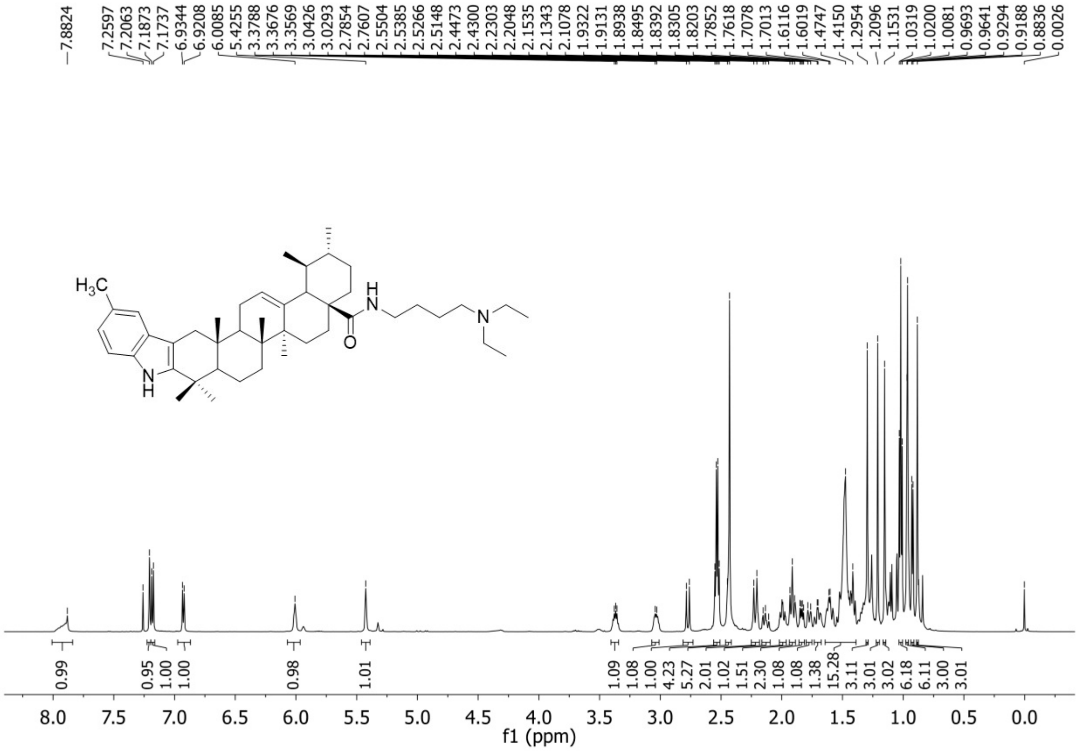

Figure S29. ^{1}H-NMR spectrum of compound **6c** (600 MHz, $CDCl_3$).

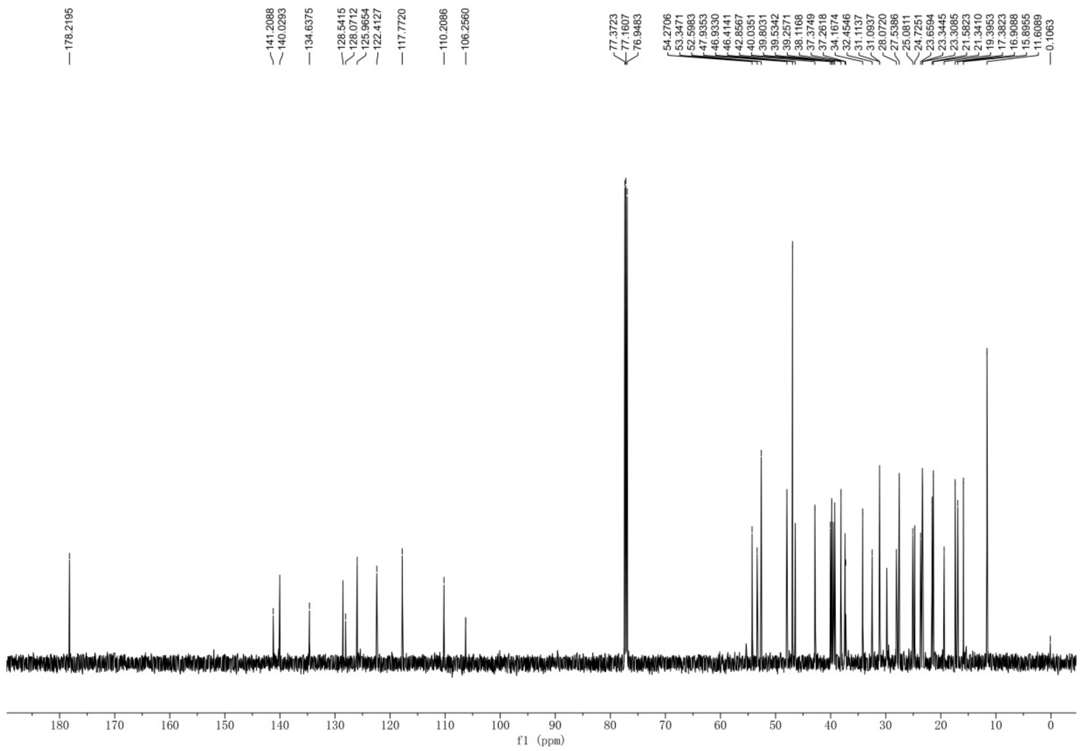

Figure S30. ^{13}C-NMR spectrum of compound **6c** (150 MHz, $CDCl_3$).

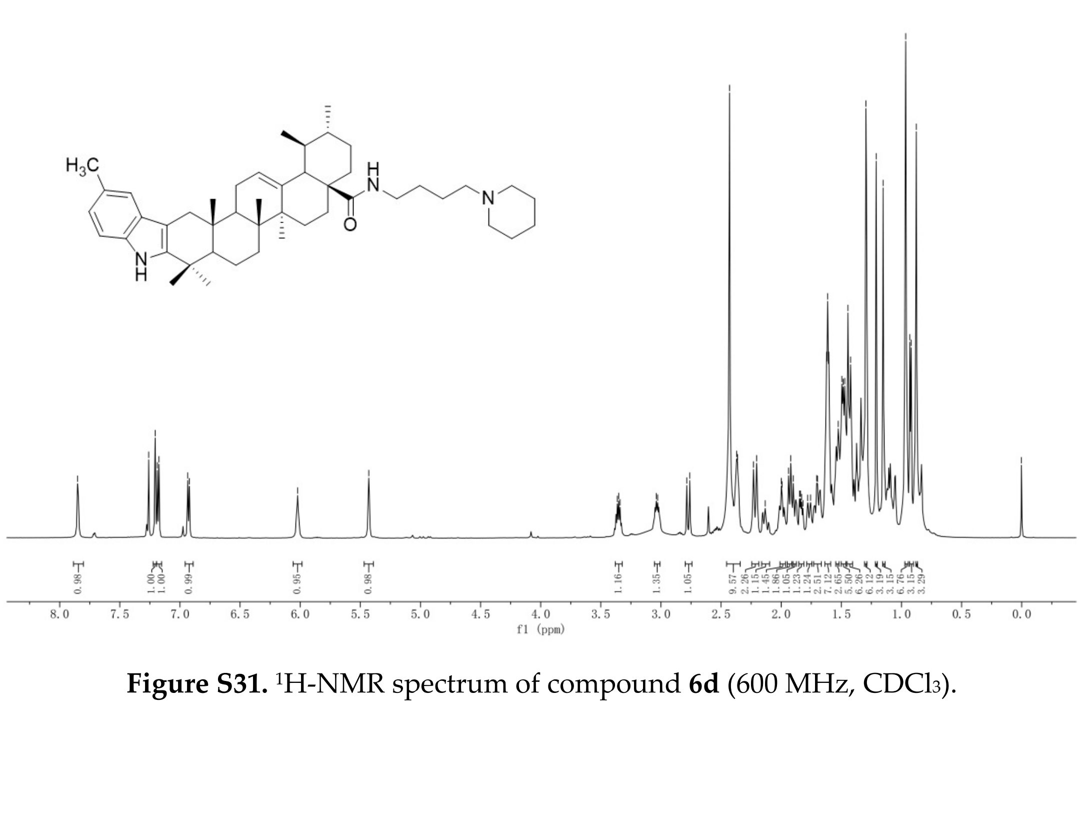

Figure S31. ^{1}H-NMR spectrum of compound **6d** (600 MHz, $CDCl_3$).

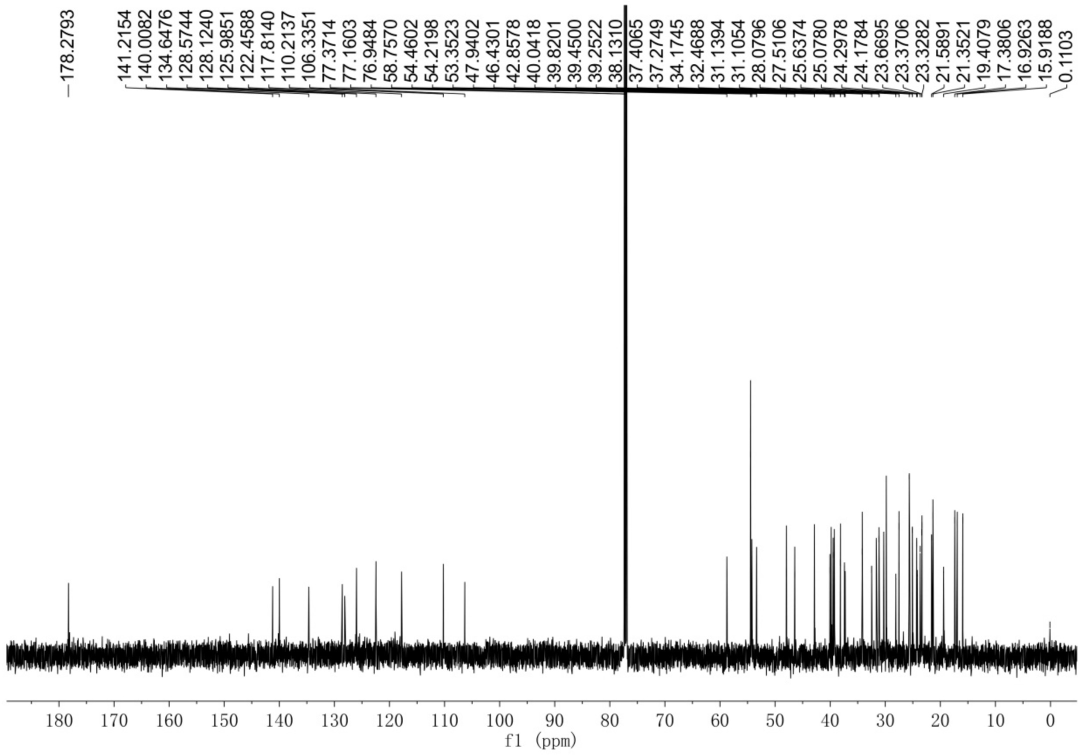

Figure S32. ^{13}C-NMR spectrum of compound **6d** (150 MHz, $CDCl_3$).

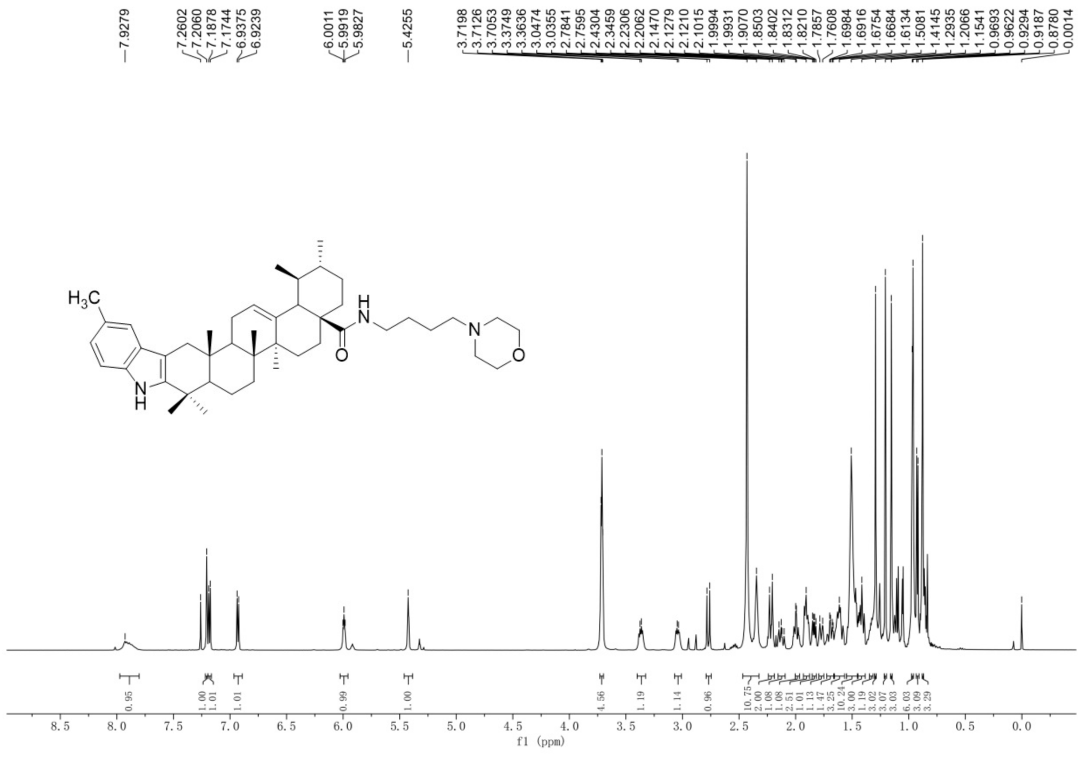

Figure S33. ^{1}H-NMR spectrum of compound **6e** (600 MHz, $CDCl_3$).

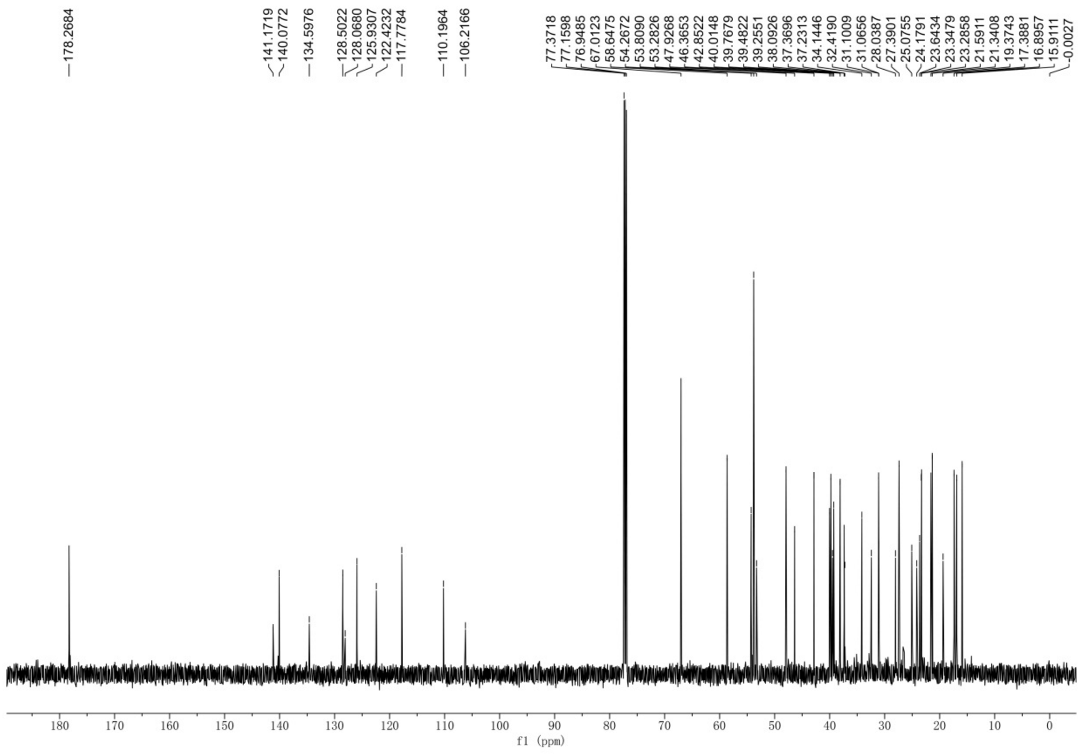

Figure S34. ^{13}C-NMR spectrum of compound **6e** (600 MHz, $CDCl_3$).

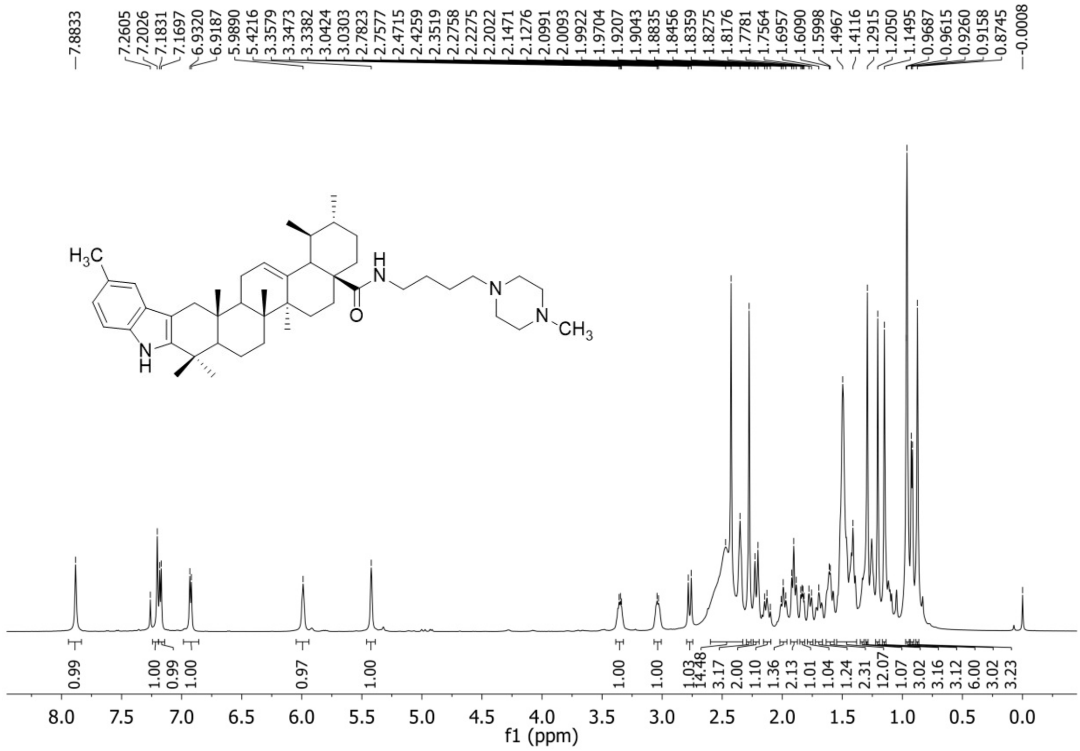

Figure S35. ^{1}H-NMR spectrum of compound **6f** (600 MHz, $CDCl_3$).

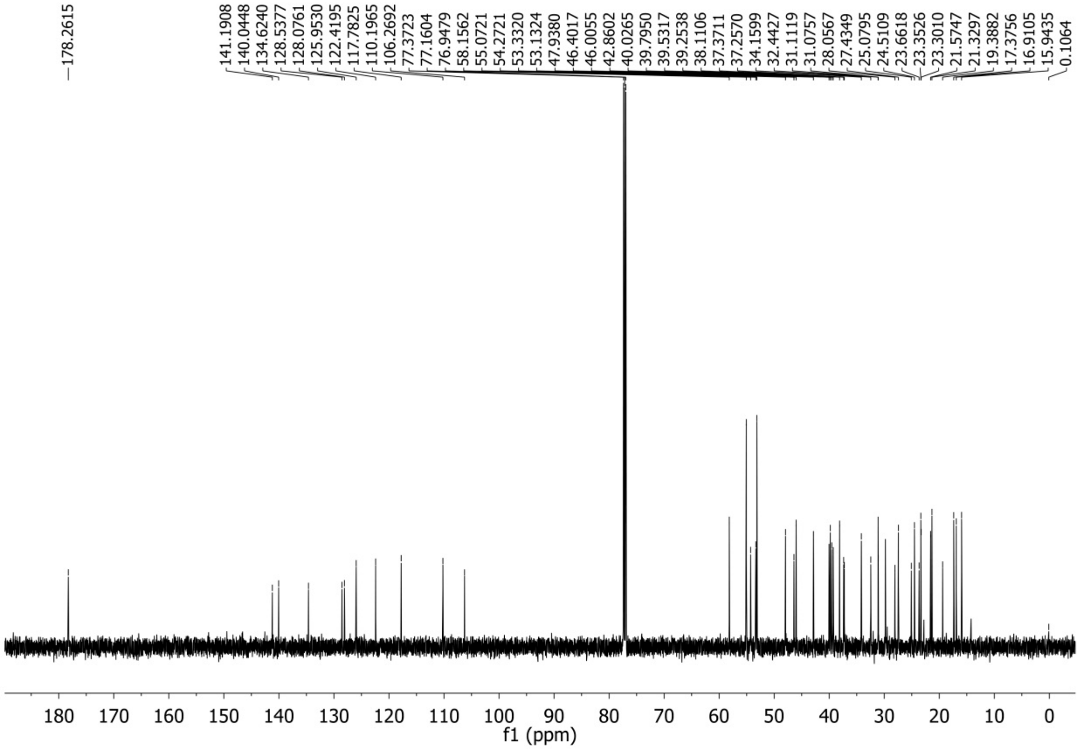

Figure S36. ^{13}C-NMR spectrum of compound **6f** (150 MHz, $CDCl_3$).

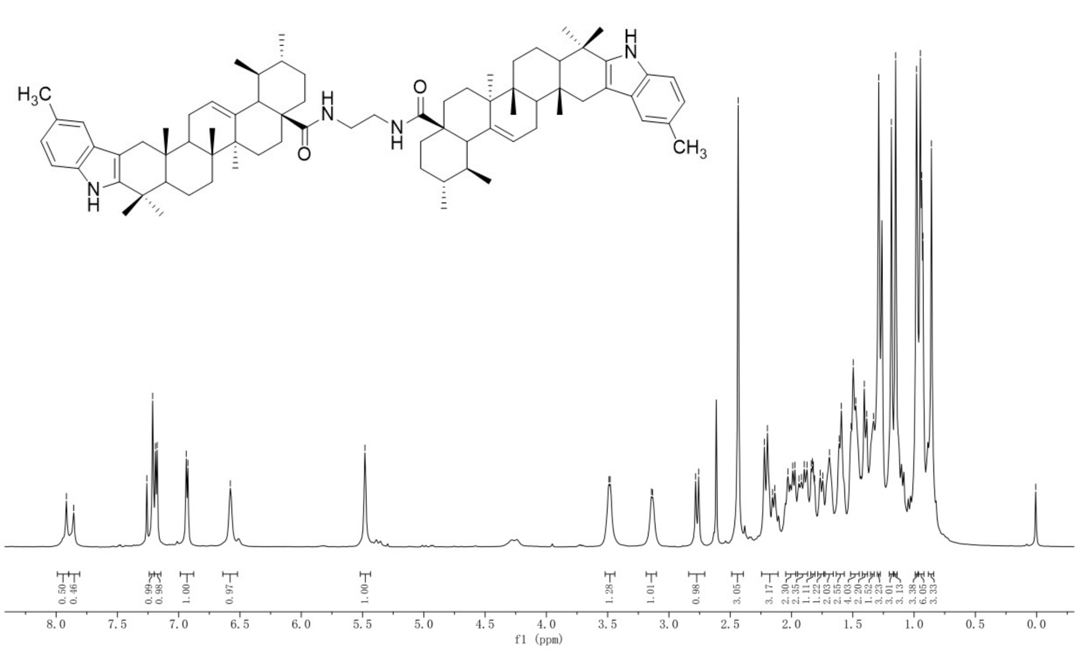

Figure S37. ^{1}H-NMR spectrum of compound **7a** (600 MHz, $CDCl_3$).

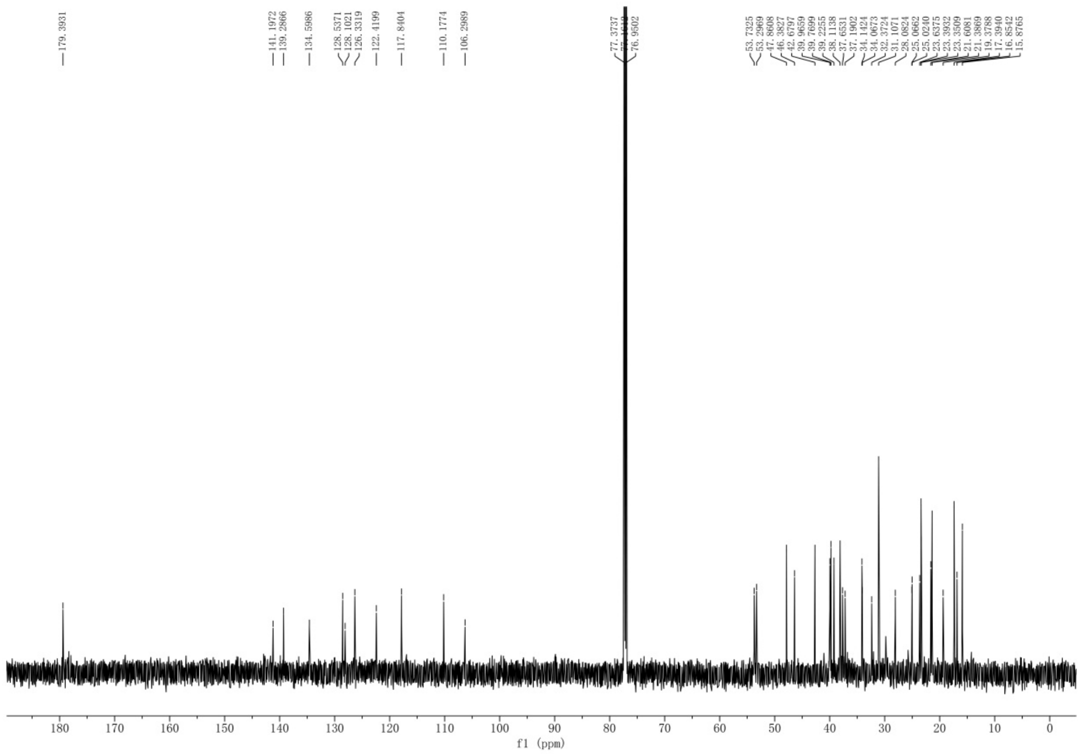

Figure S38. ^{13}C-NMR spectrum of compound **7a** (150 MHz, $CDCl_3$).

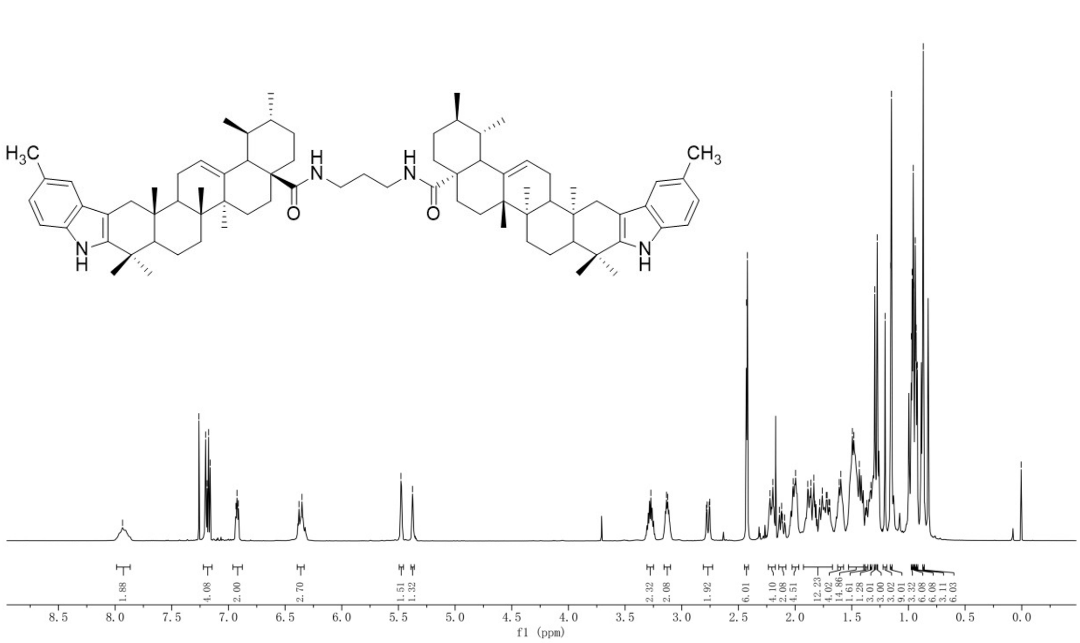

Figure S39. ^{1}H-NMR spectrum of compound **7b** (600 MHz, $CDCl_3$).

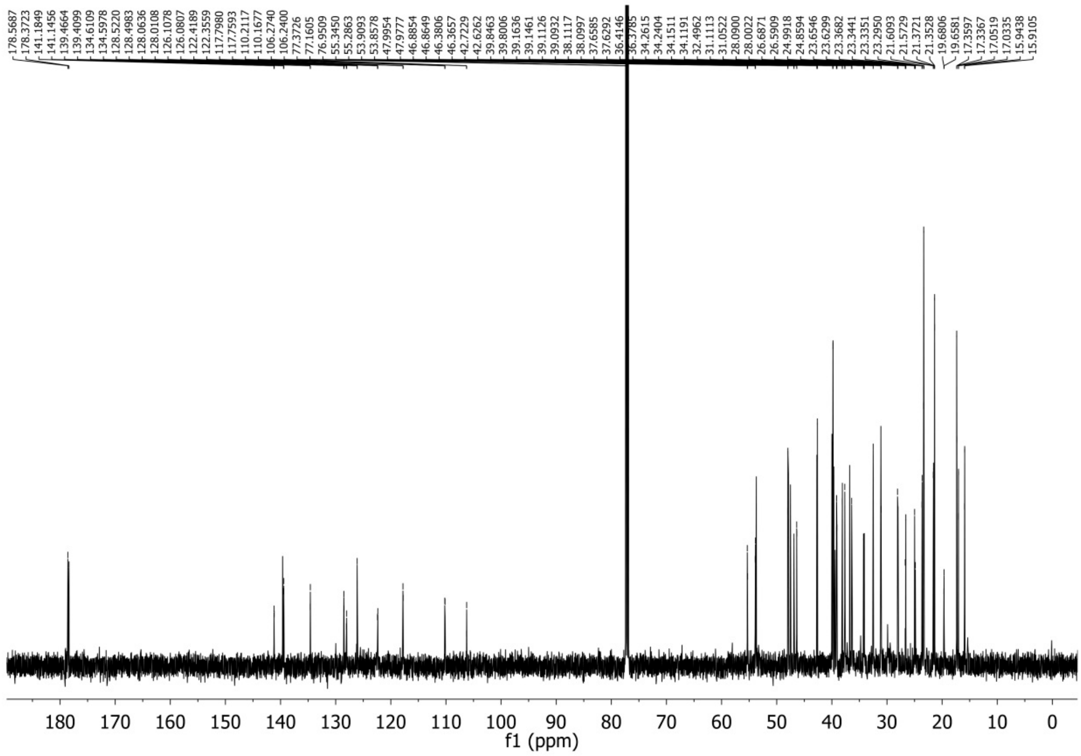

Figure S40. ^{13}C-NMR spectrum of compound **7b** (150 MHz, $CDCl_3$).

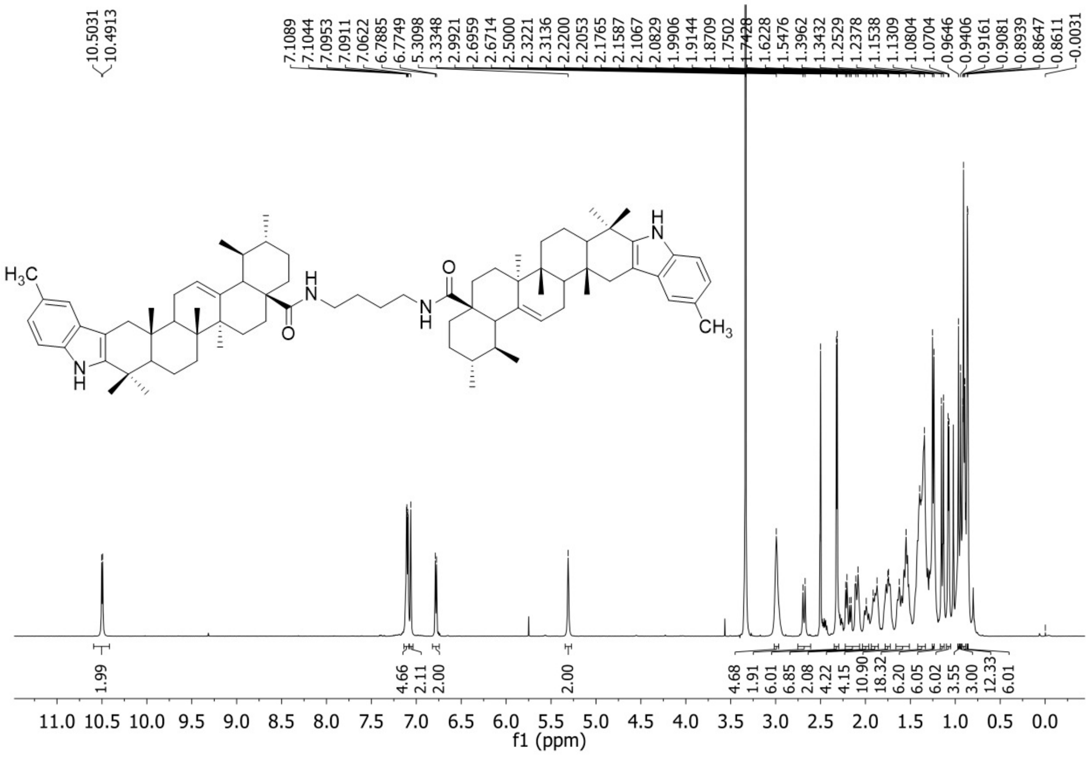

Figure S41. ^{1}H-NMR spectrum of compound **7c** (600 MHz, $CDCl_3$).

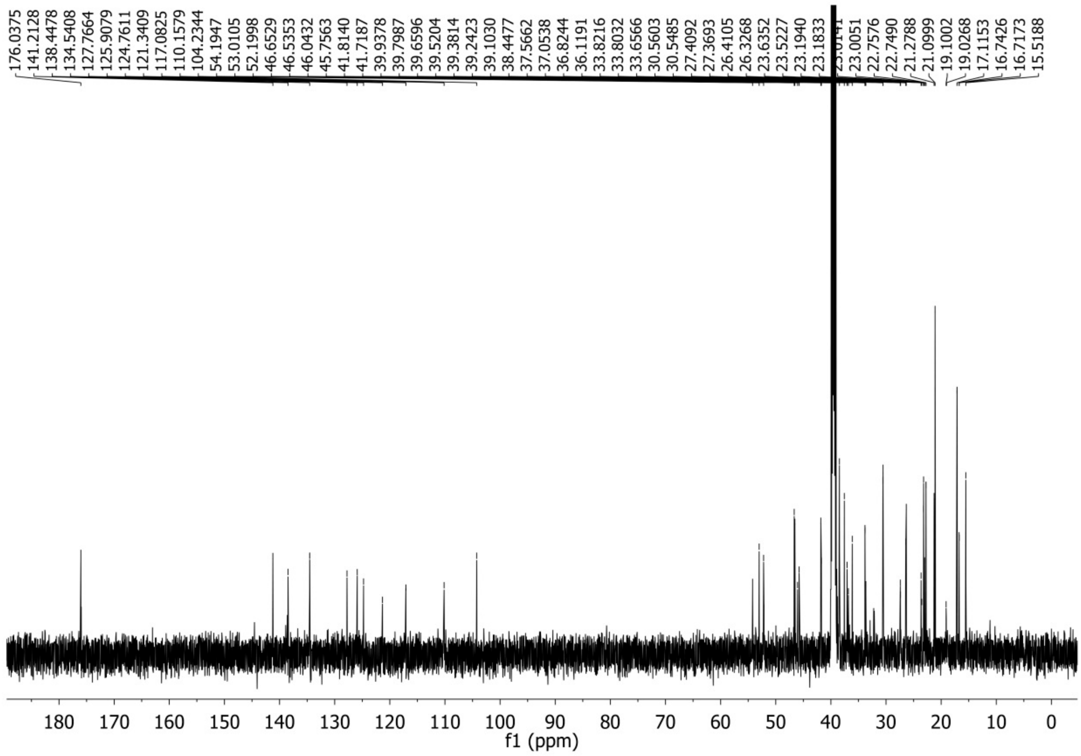

Figure S42. ^{13}C-NMR spectrum of compound **7c** (150 MHz, $CDCl_3$).
